# Scrutinizing the stability and exploring the dependence of thermoelectric properties on band structure of 3*d-*3*d* metal-based double perovskites Ba_2_FeNiO_6_ and Ba_2_CoNiO_6_

**DOI:** 10.1038/s41598-021-90027-7

**Published:** 2021-05-18

**Authors:** Shabir Ahmad Mir, Dinesh C. Gupta

**Affiliations:** Condensed Matter Theory Group, School of Studies in Physics, Jiwaji University, Gwalior, 474011 India

**Keywords:** Energy science and technology, Materials science

## Abstract

Through the conventional DFT computation, we have designed new oxide double perovskites Ba_2_FeNiO_6_ and Ba_2_CoNiO_6_. The structural and thermodynamic stabilities are predicted by optimizing the crystal structure and evaluation of enthalpy of formation, respectively. Then by using the optimized lattice constant, we have explored the different physical properties. The GGA + mBJ electronic band-structure illustrates Ba_2_FeNiO_6_ is a half-metal with 100% spin polarization at the Fermi level. While Ba_2_CoNiO_6_ shows a ferromagnetic semiconducting nature. The change in the electronic structure when Fe is replaced by Co is explained with the help of the orbital diagram and exchange interaction. The *e*_*g*_*-e*_*g*_ hybridization that happens via *O-p* states is strong because Fe–O–Ni and Co–O–Ni bond angles are strictly 180°. The narrow bandgaps in the semiconducting channels prompted us to analyze the applicability of these materials towards thermoelectric technology. Besides this, we have investigated the dependency of transport properties on electronic band structure. The semiconducting nature in Ba_2_CoNiO_6_ results in a significant ZT around 0.8 at room temperature makes it suitable for wasted-energy regeneration

## Introduction

With the passage of time and advancement in experimental techniques, new novel materials with promising capabilities to meet the challenges of current and futuristic technologies are being discovered. In the last year, room temperature superconductivity, and solar cells with 47.1% conversion efficiency at lab scale were successfully characterized^1,2^. Besides the experimental synthesis, density functional theory (DFT) based simulation is an efficient approximation method to find and explore new materials^3–5^. With the combined experimental and DFT-methods, the colossal Seebeck effect with ZT ~ 470 at around 350 K in metallic Cu_2_Se has been reported^6^. New members of series of families are being intensively designed through DFT simulations by establishing their stabilities and computation of the electronic structure along with other physical properties^7–10^. The DFT-investigation is quite helpful in predicting the nature of the materials under extreme conditions with low-cost facile methods. So, DFT examination can be considered to be the first step to define stability and predict properties of any material in a quick process before its experimental synthesis.

In the past few decades, perovskite structured materials are reported to show thought-provoking and physics-rich properties^11–15^. Oxide double perovskites (DPs) are the quaternary metal oxides with a chemical composition of A_2_BB′O_6_. The A-site occupants are mostly alkaline-earth metal (especially Ca, Sr and Ba) but any other metal that can exist in the + 3 or + 2-oxidation state with coordination number 12 can occupy the site. The most occurring oxidation states found for oxide double perovskites are A_2_^+2^(BB′)^+8^O_6_^–2^ and A_2_^+3^(BB′)^+6^O_6_^–2^ while A_2_^+1^(BB′)^+10^O_6_^–2^ configuration rarely occurs^16–18^. The B and Bʹ constituents are mostly transition/inner-transition metals. The ionic character makes these materials suitable for solid-state ionic batteries^19^. Besides this, double perovskites are known to be multifunctional showing good oxidation resistance, high Curie temperature, large spin polarization effect, high figure of merit, and other fascinating properties^20–23^. These features make double perovskites apposite for many advanced technologies. In this paper, we have designed two new DPs, Ba_2_BNiO_6_ (B = Fe and Co), having 3*d-*3*d* combination of transition metals Fe^+5^ (3*d*^3^, *t*_*2g*_^3^*e*_*g*_^0^) or Co^+5^ (3*d*^4^, *t*_*2g*_^3^*e*_*g*_^1^) and Ni^3+^ (3*d*^7^, *t*_*2g*_^6^*e*_*g*_^1^). The constituents of these materials are present in quantum quantity in the earth’s crust. Moreover, the doped form of these double perovskites and similar other perovskites has been experimentally reported. Recently, Shen et al. have synthesized Ba_2_Ni_x_Co_2−x_O_6_ (x = 0.15, 0.35, 0.55, 0.75) showing good regeneration ability^24^. Ba_2_FeCoO_6-δ_ oxygen-deficient double perovskite has been reported to be a potential electrode material for super capacitive purposes^25^. Several transition metal-based double perovskites are reported to be suitable for spintronic applications. By the use of DFT computation, Ba_2_FeMnO_6_ has been verified as a half-metallic ferromagnet with a high magnetic moment of 6*μ*_*B*_ and a large thermopower^26^. A_2_MnTaO_6_ (A = Sr, Ba) and Sr_2_FeCoO_6_ are also ferromagnetic half-metals stable in cubic *Fm-3m* structure^27–29^. In our previous studies, we have found transition metal-based perovskites La_2_CuMnO_6_ and Ba_2_CoUO_6_ are ferromagnetic half-metals while Ba_2_NiUO_6_ is a ferromagnetic semiconductor^30,31^.

The *d-d* electronic interactions in transition metal-based perovskites are strong localizing the electron orbital and spin moments^32^. The B–O–B′ bond angle in A_2_BB′O_6_ features the type of exchange interaction^33^. The B–B′ interactions of double perovskites can be gradually altered by carefully manipulating the B–O–B′ bond angle to obtain the desired properties^34,35^. If both B and B′ are magnetic, the magneto-electronic structure of such configuration is governed by B–B′ interaction. However, if one of the transition-metal ions is non-magnetic (B′ say), the properties are defined by long-distance next-next-nearest neighbor B–B exchange interactions Therefore, the choice of *d*-atoms having different electron occupancies largely influences the magneto-electronic, transport, and mechanical properties of the DPs.

## Results and discussions

The obtained results of the study and the discussion over the results are presented below under different sections.

### Structural and thermodynamic stabilities

Double perovskites (A_2_BB′O_6_) are modified form of single perovskites (ABO_3_) obtained by replacing exactly half of the B-cations with different B′-cations^17^. Ideal single perovskites are most stable in *Pm-3m* cubic structure given in Fig. S1 (Supplementary Information). However, the transition from single perovskite to double perovskite changes the prototype structure from *Pm-3m* to *Fm-3m* also shown in Fig. S1. The lattice constant in DPs is almost double of single perovskites. The B and B′ cations in *Fm-3m* structure are ordered; occupy alternative sites or even can be layer-wise ordered. However, like in the single perovskites, the size mismatch in the constituents can distort the structure. Therefore, tolerance factor (t) an empirical relation from ionic radii of constituents is widely used to predict the structure of new double perovskites^36,37^. If ‘t’ is in the range of 0.9–1, the cation sizes are perfect for the ideal structure. However, when t < 0.9 and t > 1 the constituents are in an under bonded state. Therefore, under these circumstances, BO_6_ and B′O_6_ octahedra distort to intensify bonding^31^. The values of the t-factor for Ba_2_BNiO_6_ perovskites are presented in Table 1, the evaluated values signify both materials are stable in *Fm-3m* structure. To be more convinced about the stability of Ba_2_BNiO_6_ (B = Fe and Co) in a cubic structure, we have carried out structural optimization. Ba_2_BNiO_6_ perovskites are optimized in ferromagnetic (FM), antiferromagnetic (AFM) and non-magnetic (NM) configurations with the help of spin-polarized and non-spin polarized calculations. The Birch-Murnaghan equation is used to make fit from energy-volume data and predict the optimized parameters^38,39^.

**Table 1:** Ground-state parameters of Ba_2_BNiO_6_ double perovskites namely tolerance factor ($t$); lattice constant (a); bulk modulus (B_0_) and its pressure derivative (B_0_′); optimized energy of FM phase (E_FM_); enthalpy of formation per atom (E_Form_), cohesive energy per atom (E_C_); energy difference between different magnetic phases E_FM_-E_NM_ and E_FM_-E_AFM_.

Material	*t*	a (Å)	B_0_ (GPa)	B_0_′	E_FM_ (eV)	E_C_ (eV)	E_Form_ (eV)	E_FM_-E_NM_ (eV)	E_FM_-E_AFM_ (eV)	Stable magnetic phase
Ba_2_FeNiO_6_	0.93	7.89	136.78	4.83	− 531,055.62	5.23	− 3.30	− 1.15	− 0.69	FM
Ba_2_CoNiO_6_	0.95	7.90	130.11	4.93	− 534,336.70	4.61	− 2.95	− 0.61	− 0.51	FM
Ba_2_FeMoO_6_^21^	–	8.08	144.71	4.40	–	–	–	–	–	FM
Ba_2_FeMnO_6_^26^	0.99	7.97	148.17	5.05	–	5.38	–	–	–	FM

$$E(V)={E}_{0}+\frac{9{B}_{0}{V}_{0}}{16}\left\{\left[{\left(\frac{{V}_{0}}{V}\right)}^{2/3}-1\right]\right.B{^{\prime}}_{0}+{\left[{\left(\frac{{V}_{0}}{V}\right)}^{2/3}-1\right]}^{2}\left.\left[6-4{\left(\frac{{V}_{0}}{V}\right)}^{2/3}\right]\right\}$$

**Figure 1:**
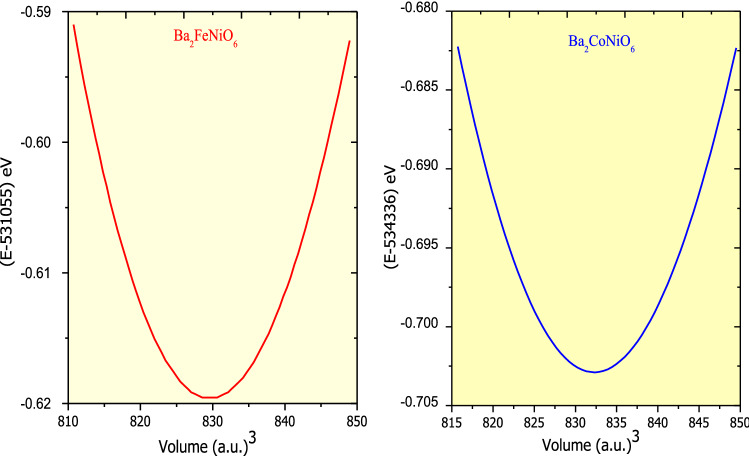
Total energy variation against volume (E-V plot) for Ba_2_BNiO_6_ (B = Fe, Co) double perovskites in the ferromagnetic ordering. The minima in the E-V optimization curve correspond to the ground state energy, the equilibrium lattice parameters are evaluated corresponding to energy minima.

Here, E_0_, V_0_, B_0,_ and B_0_′ represent energy, volume, bulk modulus, and pressure derivative of B_0_ respectively in the stress-free state. The optimization curves of the ferromagnetic phase are presented in Fig. 1. The parabolic nature of the curves authenticates the stability in a cubic structure. The optimized parameters corresponding to ground-state energy along with energy differences between possible magnetic phases are presented in Table 1. The structural parameters obtained in the present study are compatible with the already reported results of similar other double perovskites, thereby validate our results. On comparing the energy of the magnetic and non-magnetic phase mentioned in Table 1, it is evident that the ferromagnetic phase is thermodynamically most stable, it possesses the least energy.

The thermodynamic stability of these compounds is further predicted by computing the formation energy (enthalpy of formation E_form_). E_form_ remarks the difference of optimized energy of the compound and energy of its constituent atoms in their standard reference states^40^. The E_form_ for Ba_2_FeNiO_6_ perovskites is obtained through the balanced chemical equation ${4Ba}_{2}FeNi{O}_{6}\leftrightarrow 8BaO+2{Fe}_{2}{O}_{3}+4NiO+3{O}_{2}$, similarly for Ba_2_CoNiO_6_. The formation energy is therefore evaluated by using the following relation^41,42^.

$${E}_{form}^{Ba2BNiO6}={E}_{tot}^{Ba2BNiO6}-\left\{2{E}^{BaO}+\frac{{E}^{B2O3}}{2}+{E}^{NiO}+\frac{{3E}^{O2}}{4}\right\}$$

where ${E}_{tot}^{Ba2BNiO6}$ is the optimized total ground state energy, and ${E}^{BaO}$, ${E}^{B2O3}$, ${E}^{NiO}$, and ${E}^{O2}$ are total energy for BaO, Fe_2_O_3_/Co_2_O_3_, NiO, and O_2_, correspondingly. A positive E_form_ symbolizes spontaneous and unstable character, while E_form_ less than zero signifies material is thermodynamically stable. The cohesive energy (E_C_) signifies the binding energy of the constituents of a compound. E_C_ per atom can be determined using the relation^43,44^.

$${E}_{C}=\frac{\{2{E}_{aom}^{Ba}+{E}_{atom}^{B}+{E}_{atom}^{Ni}+{6E}_{atom}^{O}\}-{E}_{total}^{Ba2BNiO6}}{10}$$

where ${E}_{atom}^{Ba}$, ${E}_{atom}^{B}$, ${E}_{atom}^{Ni}$, and ${E}_{atom}^{O}$ are the bulk energies of the Ba, Fe/Co, Ni, and O-constituents, respectively. All these energies are computed by using the GGA-PBE approximation and the obtained values are given in Table 1. The obtained values of E_C_ suggest atoms are strongly held within the materials. The negative values of E_form_ for both compounds corroborate the thermodynamic stability^45^.

Besides the formation and cohesive energies, other thermodynamic properties like specific heat (*C*_*v*_), Grüneisen parameter (γ), and Debye temperature (θ_D_) are also investigated. The graphical variation of these parameters against rising temperature is specified in Fig. S2. Specific heat is the amount of energy required to raise the temperature of the material by one degree. Therefore, *C*_*v*_ represents the energy that can be stored in a material for a given temperature difference. If the temperature of that material is lowered back to the initial temperature, *C*_*v*_ is converted back to energy. So, the higher the heat capacity more could be energy stored, and likely the material acts as an efficient regenerator. The specific heat plot of the titled materials is given in Fig. S2a. The *C*_*v*_-variation with temperature indicates at low-temperature *C*_*v*_ follows T^3^ law only longwave phonon are excited in this range^46^. However, towards high temperature, all the phonons are thermally excited and *C*_*v*_ tends to Dulong limit value 3nR, R is gas constant. The discussion over variation in the γ- and θ_D_-parameters is presented in Supplementary Information.

### Electronic and magnetic behaviour

**Figure 2:**
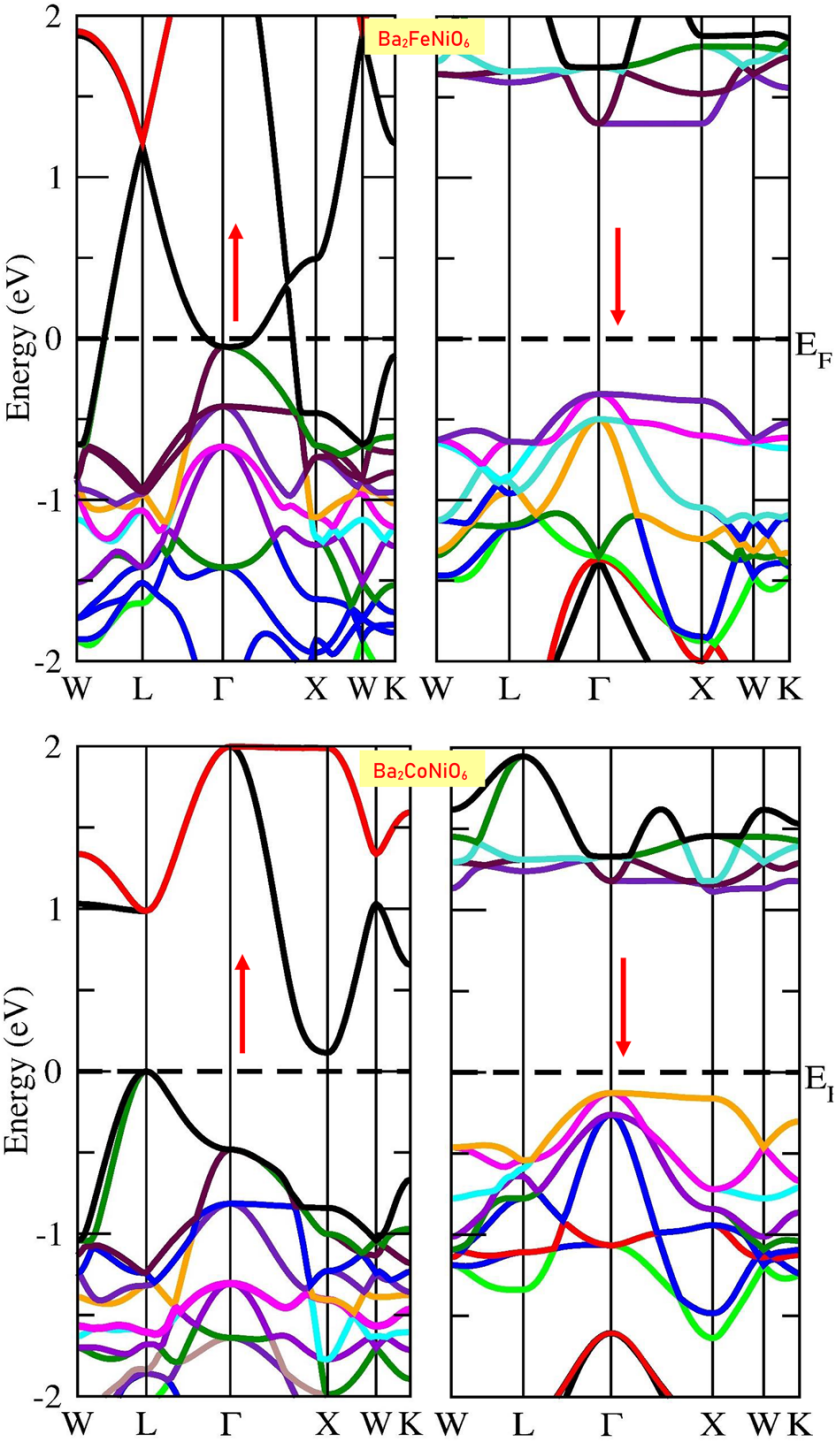
The spin-polarized band structure computed by employing GGA + mBJ approximation (the arrows are used to designate spin channels, **↑** for spin-up; **↓** for spin-down).

The band structure of Ba_2_BNiO_6_ double perovskites obtained by GGA is represented by Fig. S3. The GGA band structure indicates the presence of metallic character in these perovskites because the Fermi level passes through the bands. However, by GGA + mBJ the band structure changes effectively. The change in band structure is obvious because GGA underestimates the exchange–correlation potential and incorporation of mBJ potential sophisticatedly improves the results^47,48^. The GGA + mBJ band profiles are presented in Fig. 2, Ba_2_CoNiO_6_ is a semiconductor for both spin configurations. While Ba_2_FeNiO_6_ is half-metallic, designates metallic character in the spin-up channel and displays semiconducting behavior in the spin-down channel. The half-metallic character of Ba_2_FeNiO_6_ thereby suggests 100% spin polarization around the Fermi level^49^.

**Figure 3:**
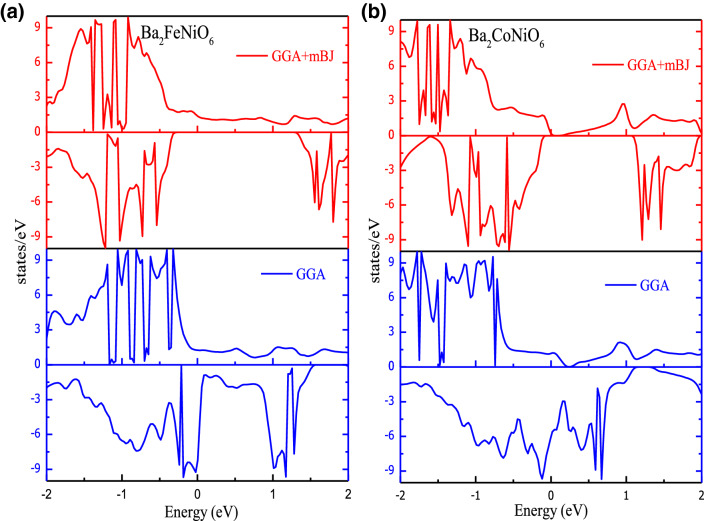
Density of states obtained by GGA and GGA + mBJ approximation (**a**) Ba_2_FeNiO_6_; (**b**) Ba_2_CoNiO_6_. High DOS peaks correspond to plethora of degenerate energy states while zero DOS symbolizes bandgap/pseudo-gap.

**Figure 4:**
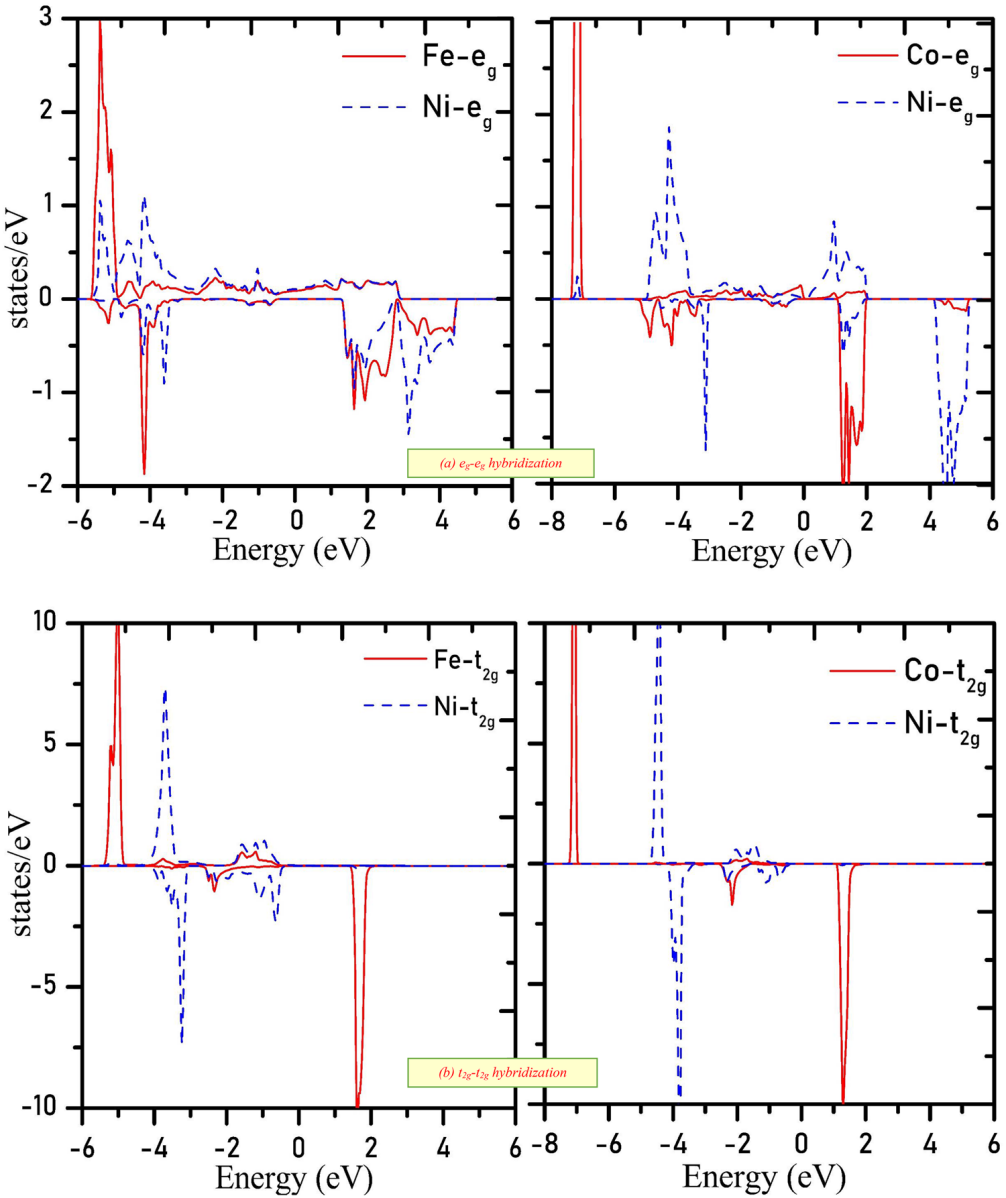
*t*_*2g*_ and *e*_*g*-_states distribution (**a**) Overlapping of *e*_*g*_*-e*_*g*_ states signify *e*_*g*_-states are hybridized; (**b**) *t*_*2g*_*-t*_*2g*_ hybridize feebly. Sharp peaks at certain energy value designate the presence of large degenerate energy states at that particular energy value.

To further discuss the electronic properties and elucidate the band structure of the materials, we have analyzed the distribution of electrons in energy states in vicinity of Fermi level. The total density of states (TDOS) obtained from GGA and GGA + mBJ methods are given in Fig. 3. With the implication of mBJ, the energy states sweep away and open the gap at the Fermi level. The high peaks in DOS indicate the presence of a plethora of energy states. Moreover, the atomic projected density of the states given in Fig. S4a,b, signifies energy states of interest are Fe-*d*, Co-*d*, Ni-*d,* and O-*p*. Figure 4a makes it certain that Fe-*t*_*2g*_ and Ni-*t*_*2g*_ states in the spin-up channel are filled give rise to DOS peaks beneath the Fermi level (set at 0 eV) for Ba_2_FeNiO_6_. The Fe-*e*_*g*_ and Ni-*e*_*g*_ states hybridize with the O-*p* states occupy the Fermi level. While in the spin-down channel, Ni-*t*_*2g*_ states are filled. But Fe-*t*_*2g*_ states are empty configure conduction band minima. The Fe*(e*_*g*_*)-*O*(p)-*Ni*(e*_*g*_*)* states even in the spin-down channel hybridize, constitute one peak below Fermi level and one above it. Also, in the Ba_2_CoNiO_6_ system, the *e*_*g*_-states of Co and Ni interact via O-*p* states. Co*(e*_*g*_*)-*O*(p)-*Ni*(e*_*g*_*)* constitute two sub-bands one in the valence band and others in the conduction band. The Co-*t*_*2g*_ states are filled in the spin-up state and empty in the down channel. Whereas, Ni-*t*_*2g*_ states are filled for both channels, illustrated in Fig. 4b. None of the state’s occupies the Fermi level in any of spin orientations suggesting the semi-conducting nature for Ba_2_CoNiO_6_. The bandgap values of Ba_2_BNiO_6_ and similar other double perovskites are summarized in Table 2.

**Table 2:** Calculated band gap (E_g_ in eV) and magnetic moment (*μ*_*B*_ per formula unit) of Ba_2_BNiO_6_ materials in comparison with similar other double perovskites.

Material	Approximation	Bandgap		Magnetic moment	Overall electronic structure
		**↑**	**↓**		
Ba_2_FeNiO_6_	GGA	–	–	4.59	M
	GGA + mBJ	–	1.66	4.0	HM
Ba_2_CoNiO_6_	GGA	–	–	5.72	M
	GGA + mBJ	0.11	1.22	5.0	SC
Ba_2_FeMoO_6_^21^	GGA	–	–	4.0	HM
Ba_2_FeMnO_6_^26^	GGA	–	0.31	7.0	HM
	GGA + U	–	3.0	7.0	HM
Sr_2_FeCoO_6_^29^	GGA + U	–	0.8	5.22	HM

*M* metallic, *SC* semiconducting, *HF* half-metallic.

### Origin of gap

**Figure 5:**
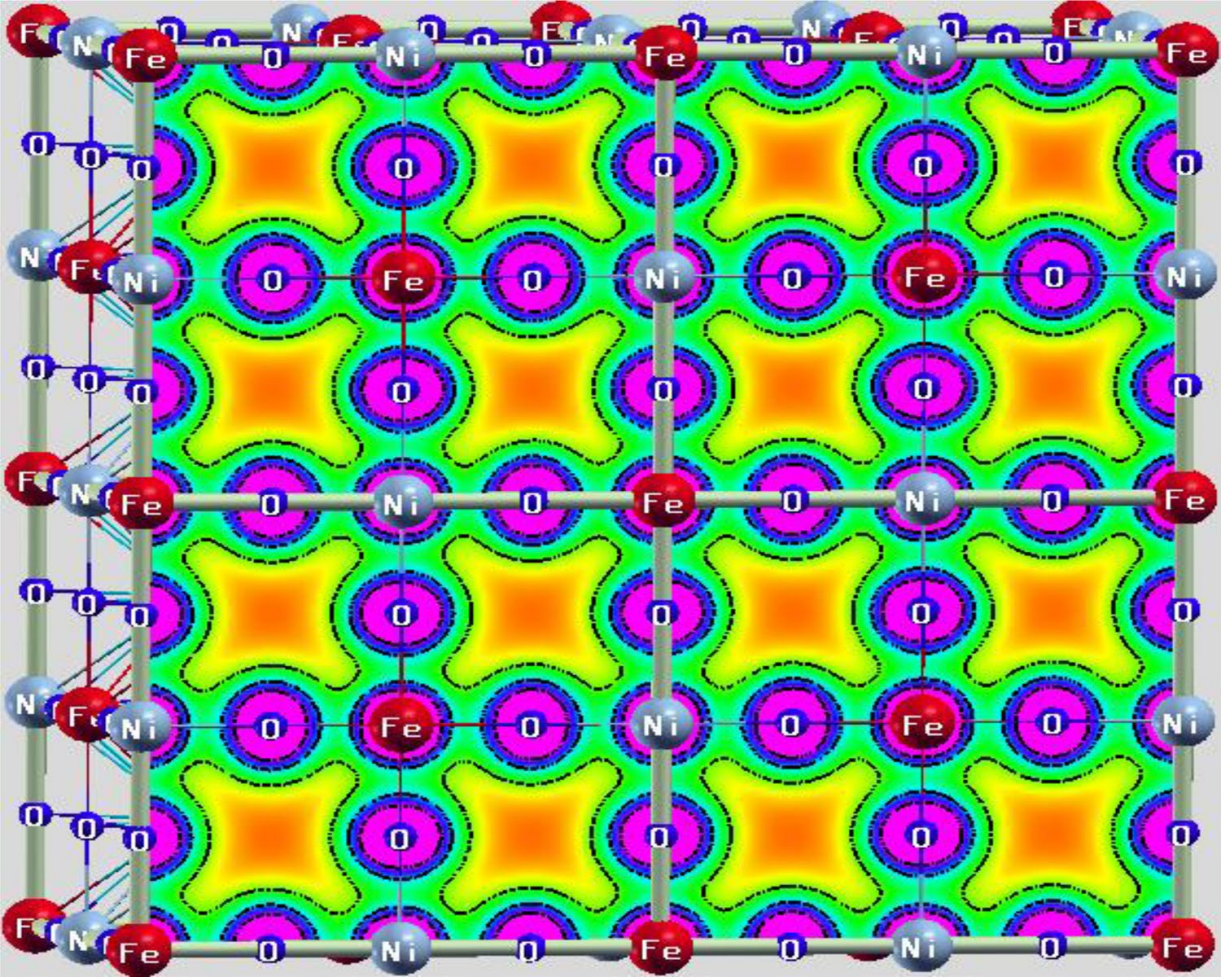
Charge density plot in (100)-plane for Ba_2_FeNiO_6_ double perovskite.

**Figure 6:**
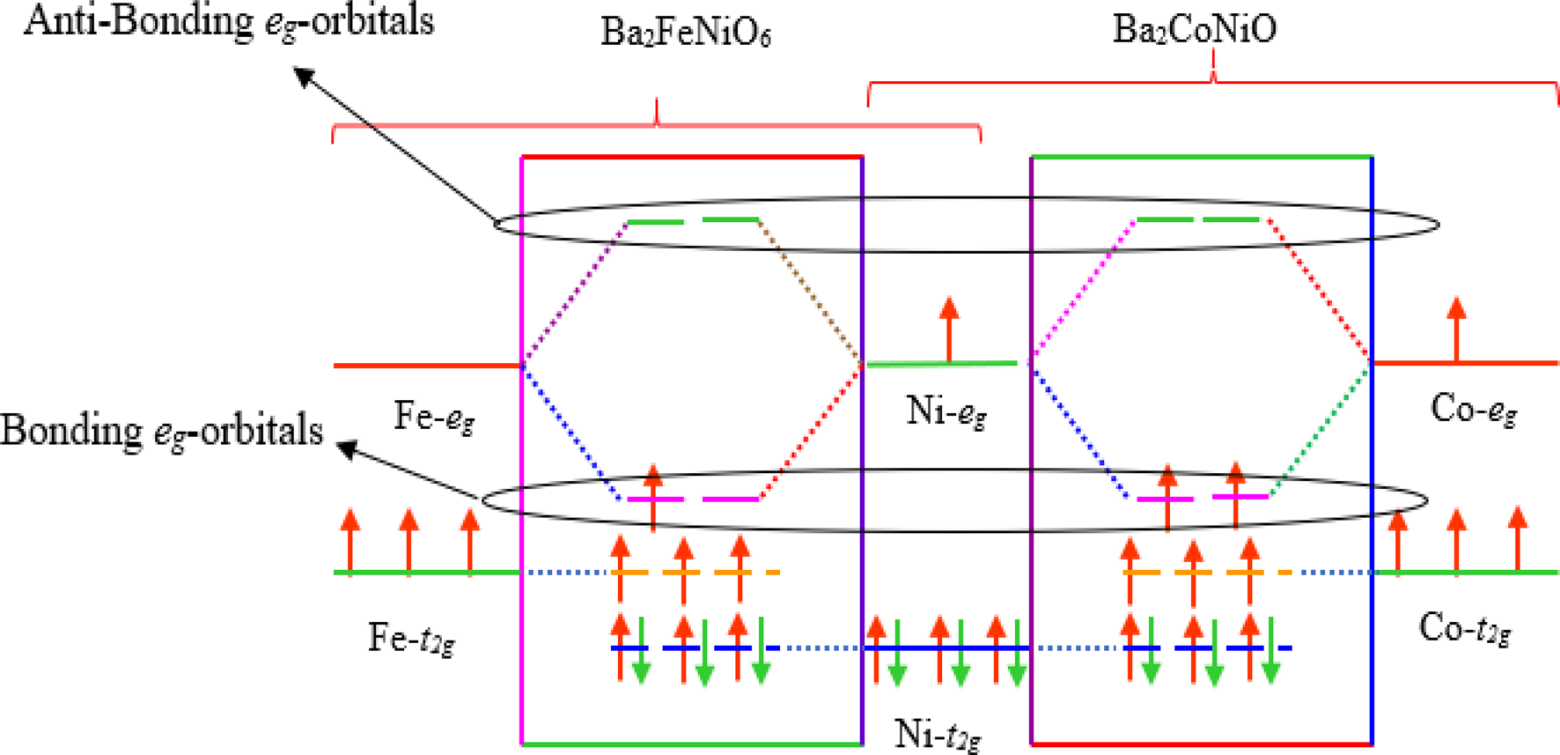
Molecular orbital splitting and formation of hybridized bonding and anti-bonding states.

The illustration of the driving mechanism for understanding the role of *e*_*g*_-states in characterizing the electronic structure is given in Fig. S5 and Figs. 5, 6. The well-known behavior of the *d*-states in the octahedral field is that they split into two separate degenerate sets *t*_*2g*_ and *e*_*g*_. The *3d*-atoms in the Ba_2_BNiO_6_ perovskites occupy alternative sites and are bonded via O-atoms. The Fe–O–Ni and Co–O–Ni bond angles are 180°. Therefore, the *e*_*g*_-states of Fe and Ni (similarly Co and Ni) in their respective materials are oriented linearly along the axes, thereby hybridize via O-*p* states symbolized in Fig. S5. However, the *t*_*2g*_ states of transition-metals have clover leaf type structure, don’t orient directly towards the orbitals of the nearest neighbor. So, *t*_*2g*_-states hybridize feebly in a lateral way with *t*_*2g*_ and *e*_*g*_-states of another transition-metal. The hybridization between *d*-states of transition atom with O-*p* can also be visualized from charge density distribution shown in Fig. 5. The overlapping of charge contours of Fe, O, and Ni evidences the possibility of *p-d* hybridization.

The orbital splitting and formation of bonding and anti-bonding *e*_*g*_-states are represented via Fig. 6. The nominal valence states maintaining the charge stability for the present set of double perovskites are Ba_2_^+2^Fe^+5^Ni^+3^O_6_^–2^ and Ba_2_^+2^Co^+5^Ni^+3^O_6_^–2^. The Fe^+5^, Co^+5^, and Ni^+3^ valence electrons are most dominant in characterizing the electronic band structure. Ni^3+^ (common cation in both perovskites) form a low spin state, six out of seven valence electrons fill *t*_*2g*_-states. The last electron enters into hybridized Fe(*e*_*g*_)*-*Ni(*e*_*g*_) and Co(*e*_*g*_)*-*Ni(*e*_*g*_) bonding states in their respective materials. Concerning the electrons of other transition-metal, in Ba_2_FeNiO_6_ the three unpaired electrons in Fe^+5^ half fill *t*_*2g*_. Implying *t*_*2g*_-states are filled in the spin-up channel, thus localized in the valence band. However, the single electron in hybridized bonding states of (Fe)*e*_*g*_-(Ni)*e*_*g*_ partially fills them. Therefore, (Fe)*e*_*g*_-(Ni)*e*_*g*_ occupy the Fermi level of the spin-up channel. The Co^5+^ in Ba_2_CoNiO_6_ forms a high spin state, three valence electrons out of four fill spin-up *t*_*2g*_-states. While the fourth electron enters to Co(*e*_*g*_)*-*Ni(*e*_*g*_) bonding states. Therefore, one electron from Co^5+^ and the other from Ni^3+^ half fill the hybridized (Co)*e*_*g*_-(Ni)*e*_*g*_ bonding states, bring them down to the valence band and open the gap at the Fermi level of the spin-up channel. In the spin-down channel of both materials, only Ni-*t*_*2g*_ states are filled. While all other *d*-states are empty, reside in the conduction band with a gap at the Fermi level. The overall number of unpaired electrons in FeNi- and CoNi-perovskites is 4 and 5, respectively. Due to which the total magnetic moment is 4*μ*_*B*_ and 5*μ*_*B*_ in Ba_2_FeNiO_6_ and Ba_2_CoNiO_6_, respectively, following the Slater-Pauling rule^50^.

### Thermoelectric properties

**Figure 7:**
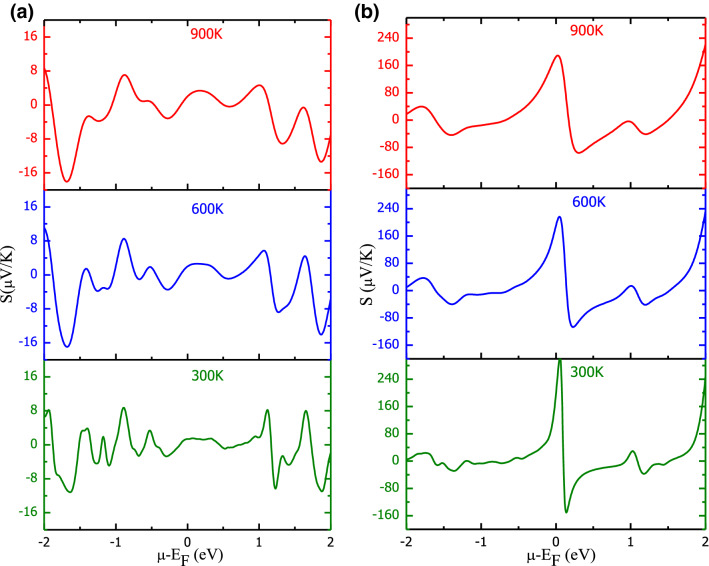
Total Seebeck coefficient as a function of chemical potential and temperature: (**a**) Ba_2_FeNiO_6_; (**b**) Ba_2_CoNiO_6_.

**Figure 8:**
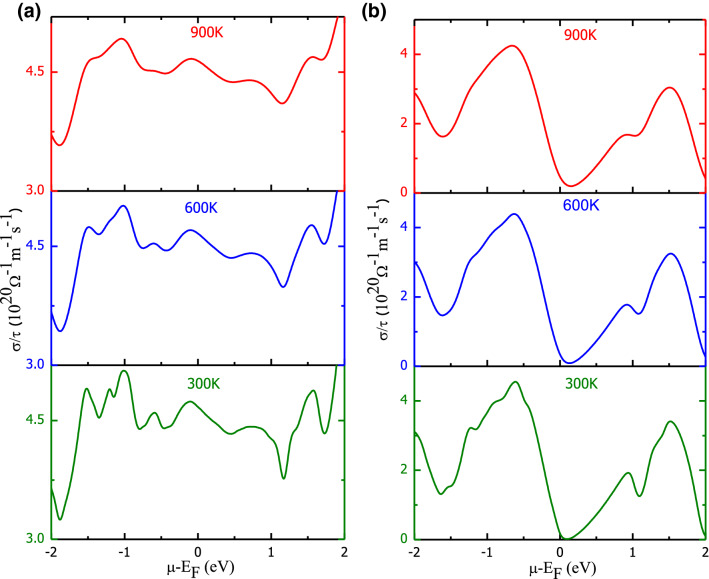
Variation in total electronic conductivity coefficient over relaxation time against chemical potential and temperature: (**a**) Ba_2_FeNiO_6_; (**b**) Ba_2_CoNiO_6_.

**Figure 9:**
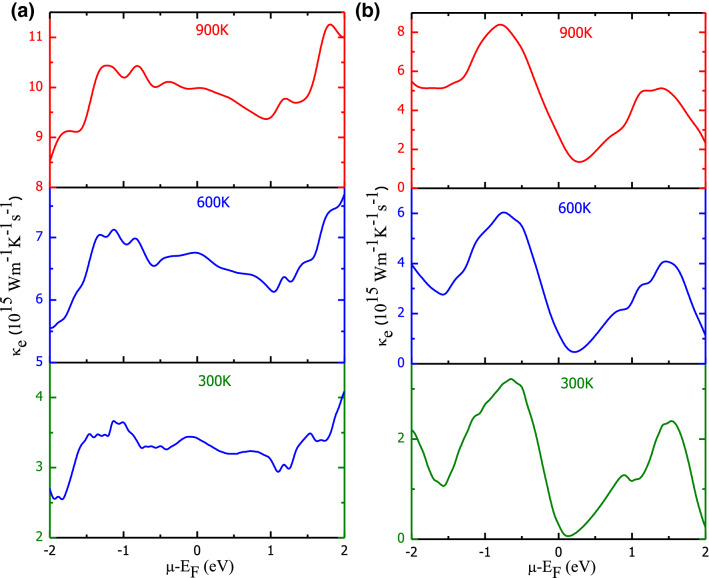
Variation in total electronic thermal conductivity with chemical potential and temperature: (**a**) Ba_2_FeNiO_6_; (**b**) Ba_2_CoNiO_6_.

**Figure 10:**
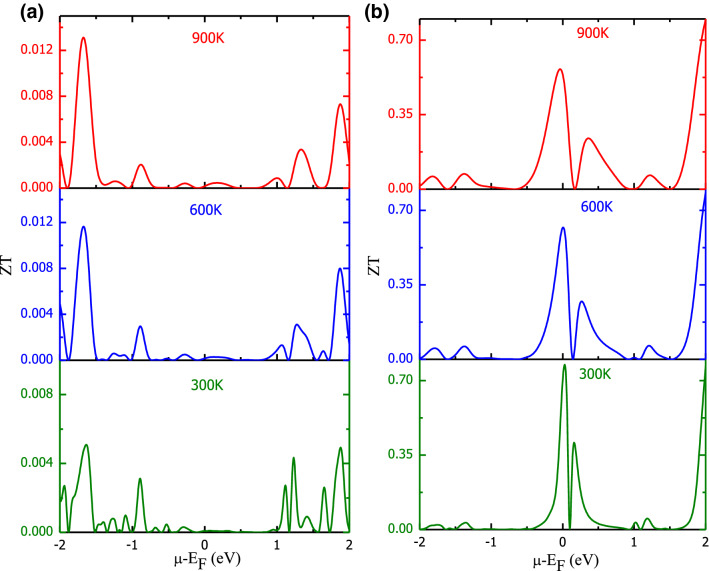
Behaviour of ZT without accounting lattice thermal conductivity with changing chemical potential at different temperatures: (**a**) Ba_2_FeNiO_6_; (**b**) Ba_2_CoNiO_6_.

The thermoelectric response of Ba_2_BNiO_6_ materials has been analyzed by studying the behavioral variation in transport parameters with chemical potential at different temperatures. The total conductivity (σ) and Seebeck coefficient (S) are defined with the help of two current model. According to which; ${\sigma }_{tot}= {\sigma }_{\uparrow }+{\sigma }_{\downarrow }$ and ${S}_{tot}=({S}_{\uparrow }{\sigma }_{\uparrow }+{S}_{\downarrow }{\sigma }_{\downarrow })/{\sigma }_{tot}$; where arrows designate respective spin-channels^51–53^. The variation in total Seebeck coefficient, total conductivities along with the figure of merit (ZT) is presented in Figs. 7, 8, 9, 10. The dissimilar electronic filling in spin channels of magnetic materials suggests electrons in these spin channels experience different driving forces thereby exhibit variant scattering rates. So, it is natural for titled materials that spin-up and spin-down electrons have dissimilar transport behavior, which is discussed in Supplementary Information. The total Seebeck coefficient of FeNi-based double perovskite has several kinks, presented in Fig. 7a, nevertheless, the peak values remain low. However, CoNi-based perovskite shows a high Seebeck coefficient with main peaks centered on either side of the Fermi level, see Fig. 7b. On comparing the magnitude of S, Ba_2_CoNiO_6_ shows higher thermopower than Ba_2_FeNiO6. This is because of the semiconducting nature in both spin channels of Ba_2_CoNiO_6_ compared to the metallic nature in one spin channel of Ba_2_FeNiO_6_. With the increase in temperature the peak values of |S| decreases gradually. The decreasing behavior can be credited to the smearing of energy bands. The variation in the total electronic conductivity coefficient over relaxation time (σ/τ) is presented in Fig. 8a,b. The electronic conductivity variation with temperature is gentle, but with the chemical potential, it is steep. Corresponding to the forbidden gap in band structure conductivity has to be zero, therefore vanishing conductivity at 0 eV for CoNi-indicate the semiconducting nature. However, with the rise in temperature conductivity increases slightly at the Fermi level it is because of energy band smearing. On the other side, although FeNi-based perovskite is a half-metal, total conductivity depicts the metallic-like behavior. The conductivity peaks at the Fermi level should be wholly contributed by the spin-up channel electrons. To confirm this, we have plotted variation in conductivity against the chemical potential of spin up and down channels separately, shown in Fig. S6 and Fig. S7.

The chemical potential (μ) predominantly decides which carriers (electrons and holes) take part in transport phenomenon^54^. So, the transport properties, both conductivities, and Seebeck coefficient, extensively depend on the value of chemical potential. Also, from Figs. 7 and 8 it is conclusive that at a fixed temperature, a rapid variation in the electrical conductivity and Seebeck coefficient occurs with a change in chemical potential. The electronic band profile has parabolic degenerate bands in the vicinity of the Fermi level with different *k*-dispersion. To correlate the influence of electronic properties on the transport properties, we have plotted the Seebeck coefficient, electrical conductivity at 300 K together with volumetric DOS for Ba_2_CoNiO_6_. The comparative variation in these parameters within the vicinity of the Fermi level is presented in Fig. S8. The large Seebeck coefficient around the Fermi level can be ascribed to the vanishing density of states^55^. As we move away from the Fermi level, the DOS peaks increase result in a decrease in the Seebeck coefficient. Corresponding to every peak in DOS there is a peak in the conductivity. While zero conductivity can be seen against bandgap/pseudo-gap. The highly populated DOS regions show a low Seebeck coefficient and high conductivity. Thereby, confirming that magnitude of transport properties is directly related to the behavior of energy levels close to the Fermi level. With the increase in temperature, energy band smear and some states that were empty at T = 0 K are now filled because the electrons changeover states due to gain in thermal energy. As the band smearing happens, the Seebeck coefficient at high DOS regions increases while conductivity decreases. The reverse is the case for the low/zero DOS populated energies. The effect of carrier concentration on the magnitude of S- and σ/τ- is described in Supplementary Information. The electronic thermal conductivity over relaxation time (κ_e_/τ) presented in Fig. 9a,b also demonstrates a similar kind of behavior against chemical potential variation as shown by σ/τ. However, the thermal conductivity increases abruptly with temperature compared to electric conductivity. Also, FeNi- shows higher conducting capacity compared to CoNi-perovskite. It because of the half-metallic electronic profile.

The figure of merit (ZT) is a prime factor that characterizes the desirability of materials towards thermoelectric applicability. The variation in ZT with chemical potential at different temperatures is illustrated via Fig. 10a,b. The ZT-value of FeNi-based perovskite is very low, the highest value goes around 0.013 that too away from the Fermi level. Although Ba_2_FeNiO_6_ has 100% spin polarization at the Fermi level, however, the thermoelectric results are poor mostly due to high thermal conductivity and low Seebeck coefficient. The overall transport properties of Ba_2_FeNiO_6_ have effectively dominant character of the metallic channel. The spin split ZT provided in the Supplementary Information, wherefrom it can be seen spin-down channel of FeNi-based having semiconducting nature has ZT ~ 1. However, CoNi-based perovskite offers significant peaks in ZT on either side of the Fermi level with prominent peaks having ZT ~ 0.8, slightly lower than the reported value for Ba_2_FeMoO_6_ ~ 0.99^21^. The high value of ZT in Ba_2_CoNiO_6_ can be attributed to the semiconducting band profile. But with temperature rise, the peak values drop down because of the combined effects of the decrease in Seebeck value and increase in thermal conductivity.

### Computation methodology

All the calculations in the present study have been carried out with the help of the *Wien2k simulation code* in its full potential formalism^56^. The ground-state electron density for the perovskites is obtained with help of the Kohn–Sham (K-S) equation; wherein the exchange and correlation interaction have been estimated by well-known *generalized gradient approximation* under Perdew, Burke, and Ernzerhof parameterization^57^. Moreover, we have facilitated GGA by the *modified Becke-Johnson* (mBJ) potential to be more accurate^58^. The more detailed information regarding the parameters fitted for the present study is mentioned in Supplementary Information. The transport applicability is explored with the help of *Boltztrap code*^59^, wherein the Boltzmann equation is solved under the approximation of constant relaxation time (τ = 0.5 × 10^-14^ s). The relaxation time is a variable parameter, the magnitude of ‘τ’ undoubtedly affects the transport features. However, if the variations in ‘τ’ are gentle on the energy scale, then constant relaxation time approximation works efficiently^60,61^. The thermodynamic properties are obtained with the help of *Gibbs2 package* integrated with *Wien2k code*^62^.

## Conclusion

In the presented work, the structural stability along with electronic and transport properties are investigated using the FP-LAPW scheme with GGA and GGA + mBJ approximations. The GGA + mBJ functional employed is found to produce the half-metallic charter for Ba_2_FeNiO_6_ and semiconducting nature for Ba_2_CoNiO_6_. The possible strong exchange interaction occurs between the *e*_*g*_-states of Fe (Co) and *e*_*g*_-states of Ni because Fe-O-Ni and Co–O-Ni bond angles are 180°. The *t*_*2g*_-orbitals of transition metals are less involved in the exchange interaction. However, the finite overlap between these orbitals can be introduced by tuning the bond angles and lattice parameters as a consequence of mismatching the cations, thereby distorting the octahedra. The resultant transport properties suggest Ba_2_FeNiO_6_ conduct like that of metal while Ba_2_CoNiO_6_ conductivity happens only after carries gain sufficient thermal energy. The ZT for Ba_2_CoNiO_6_ turns out to be ~ 0.8 at room temperature. The 100% spin polarization at the Fermi level indicates FeNi-based perovskite can be used for spintronic applications and high ZT supports Ba_2_CoNiO_6_ to be used as a thermoelectric material.

## Supplementary Information

Supplementary Information.

## Data Availability

The datasets generated by the computation and thereafter analyzed would be available from Mr. Shabir Ahmad Mir on reasonable request.
