# Supplementary material for: Scrutinizing the stability and exploring the dependence of thermoelectric properties on band structure of 3d-3d metal-based double perovskites Ba2FeNiO6 and Ba2CoNiO6
Source: Sci Rep. 2021 May 18;11:10506. doi: 10.1038/s41598-021-90027-7 (PMC8131693; doi:10.1038/s41598-021-90027-7)
Supplement: Supplementary file 1 — Supplementary Information. [file 41598_2021_90027_MOESM1_ESM.docx]

**Supplementary Information**

**Scrutinizing the Stability and Exploring the Dependence of Thermoelectric Properties on Band Structure of 3*d-*3*d* Metal-Based Double Perovskites Ba_2_FeNiO_6_ and Ba_2_CoNiO_6_**

**Shabir Ahmad Mir****^#^ and Dinesh C. Gupta^*^**

Condensed Matter Theory Group, School of Studies in Physics,

Jiwaji University Gwalior- 474011 (INDIA)

*E-mail: mirshabir7500@gmail.com***^#^***;* [*sosfizix@gmail.com*](mailto:sosfizix@gmail.com)***^*^***

**Methods**

The linearized augmented plane-wave basis set with $l_{max} = 10$ and $R_{MT}K_{max}= 7$ (where $R_{MT}$ is the radius of the smallest sphere and $K_{max}$ represent maximum *k-*value) has been used. The Brillouin zone (BZ) is disintegrated into a dense *k*-mesh of 1000 integration points crucial for the convergence of the results. The iterations for solving the Kohn-Sham are allowed till the charge for successive cycles converges up to 0.0001e order and energy up to 0.001eV, respectively.

The onsite Coulomb repulsion in transition metal-based materials is strong enough to localize the electrons. However, GGA parameterization does not precisely define the exchange potential and therefore does not predict the required splitting of *d*-states for such materials. The GGA needs to be supported to explain the electronic structure and magnetic properties more sophisticatedly. There are several alternatives like the inclusion of Hubbard ^[1]^ (U_eff_ = U-J) or mBJ potentials to GGA or even hybrid functions like B3LYP^[2]^ can be used. Here, we have used the used GGA+mBJ scheme to define electronic properties more efficiently. The choice of choosing mBJ over other alternatives is that it is a facile and purely *ab-initio* technique working very well for systems with *d*-electrons ^[3,4]^. The unit cell volume is shared among muffin tin spheres surrounding the motif (with radii $R_{MT}$) and interstitial space. The Seebeck (S), electrical conductivity (σ) and electronic thermal conductivity (κ_e_) coefficients can be evaluated by using relation relations reported else ^[5,6]^;

$$S=\frac{ek_{B}}{\sigma}\int\left(-\frac{\partial f_{0}}{\partial\varepsilon}\right)\frac{\varepsilon-\mu}{k_{B}T}\Xi\left(\varepsilon\right)d\varepsilon$$

$\sigma=e^{2}\int\left(-\frac{\partial f_{0}}{\partial\varepsilon}\right)\Xi\left(\varepsilon\right)d\varepsilon$,

$\kappa_{e}=k_{B}^{2}T\int\left(-\frac{\partial f_{0}}{\partial\varepsilon}\right)\left(\frac{\varepsilon-\mu}{k_{B}T}\right)^{2}\Xi\left(\varepsilon\right)d\varepsilon$.

Here, $\Xi(\varepsilon)$ is a transport distribution function given by $\Xi^{\alpha,\beta}(\varepsilon)=\sum_{k} \delta(\varepsilon-\varepsilon_{k})v_{k}^{\alpha}v_{k}^{\beta}\tau_{k}$. where $v_{k}^{\alpha}$ depict *α^th^* component of the group velocity having wave vector *k*. The *k*-mesh was increased to 100000-*k* points to have better output.

S**tructural Properties**





**a**


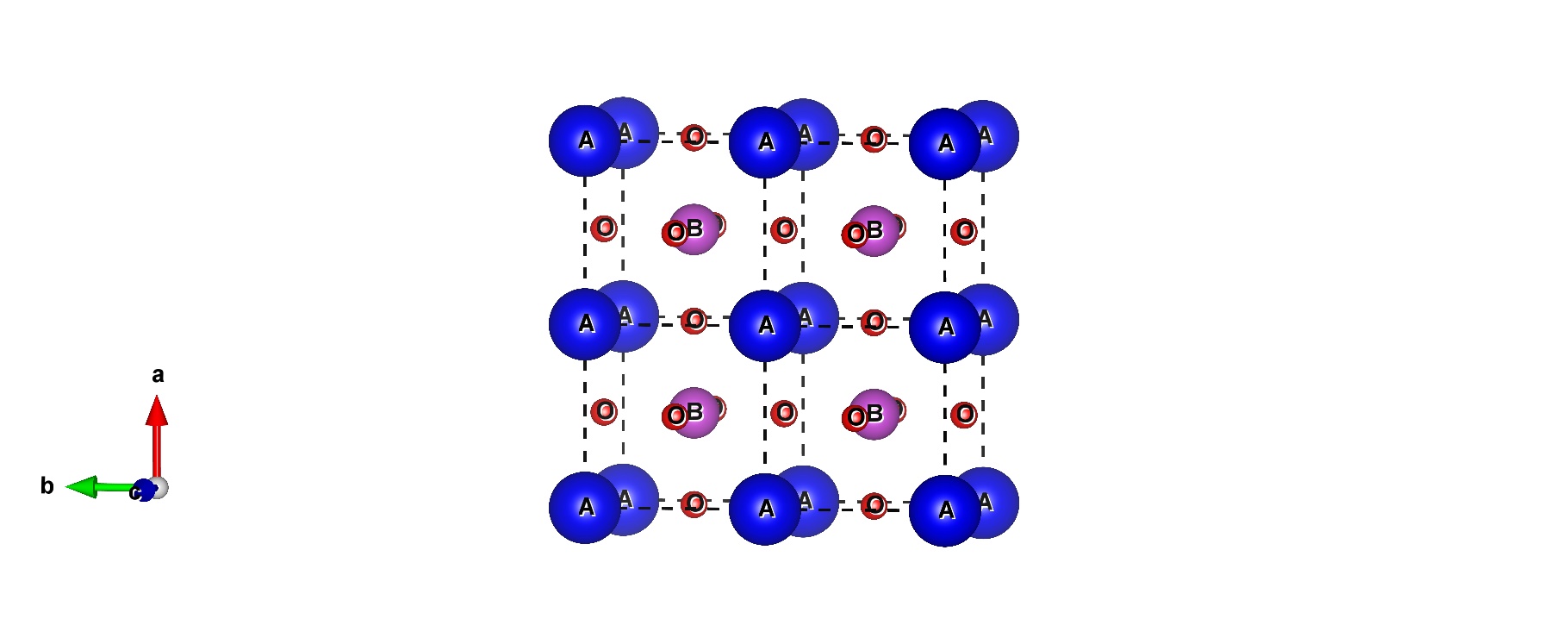

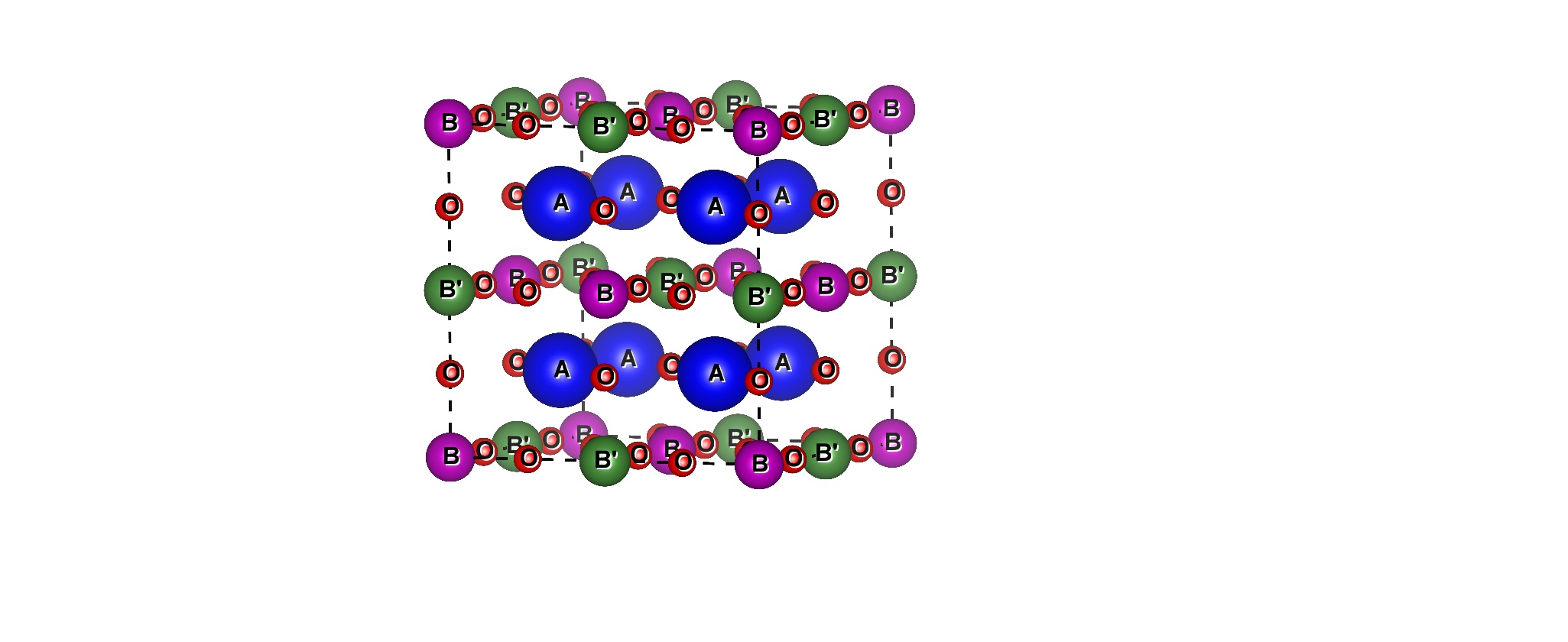


A_2_BB’O_6_-type structure

Structural transformation from ideal perovskite to ideal double perovskite

ABO_3_-type structure

AO_12_-Cage





BO_6_-Octahedra

**b**


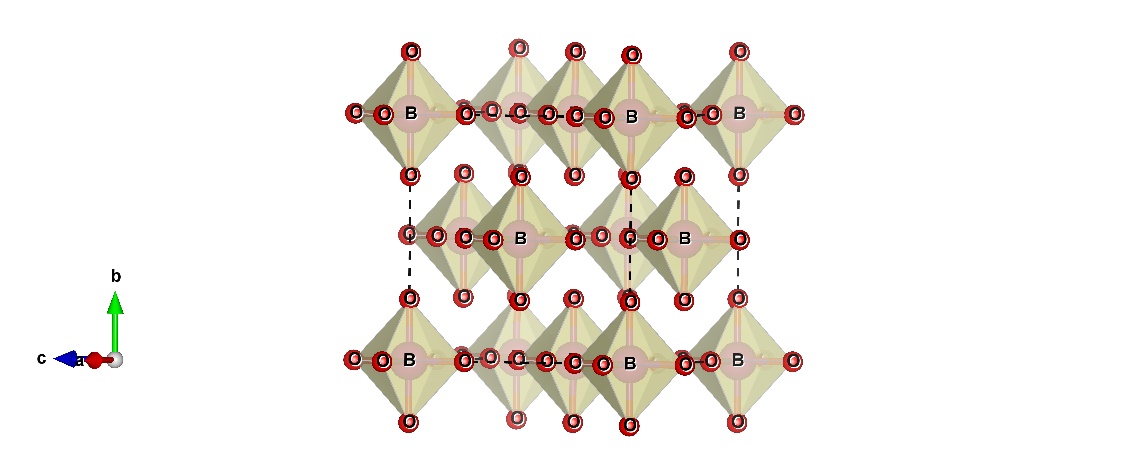

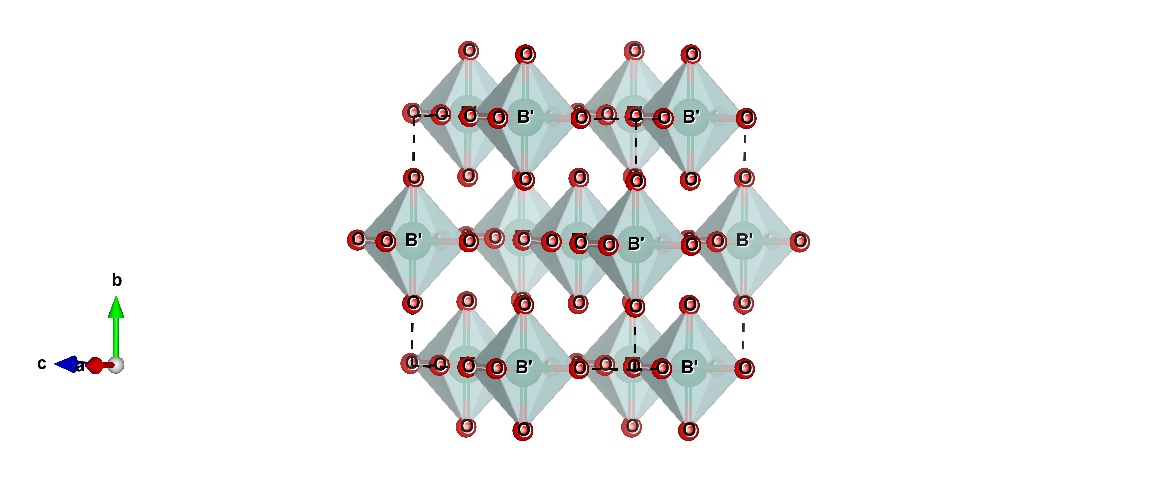

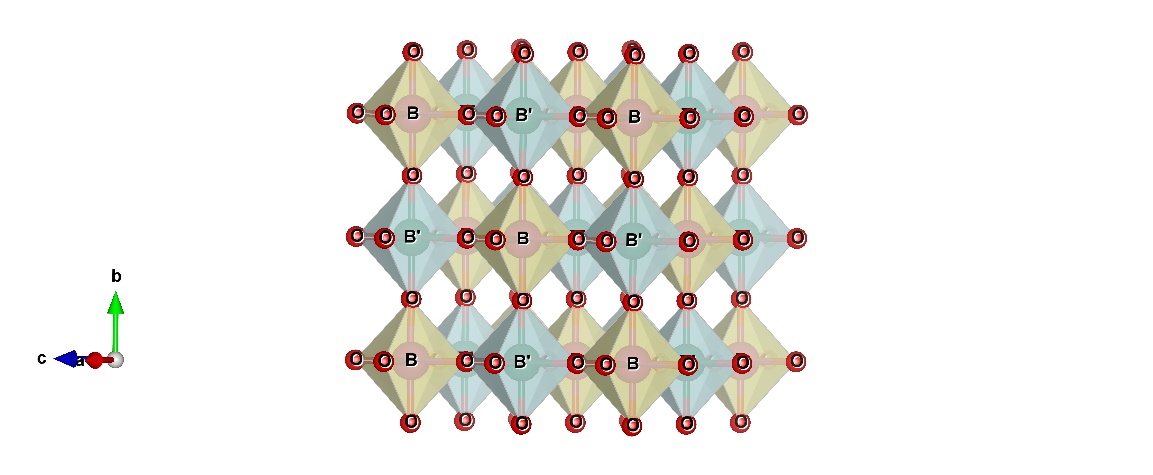


Octahedral ordering in A_2_BB’O_6_

B’O_6_-Octahedra

BO_6_-Octahedra

**+**

**Fig. S1:** (a) Ideal crystal structure of simple perovskites and double perovskites. Within the structure B (B’)-atoms are filled inside the octahedra and A-is surrounded by 12-oxygen atoms. (b) The crystal structure of A_2_BB’O_6_ is composed of two ordered sub-structure BO_6_ and B’O_6_ extending over 3D with A-occupying inter-octahedral voids.

**Thermodynamic Properties**


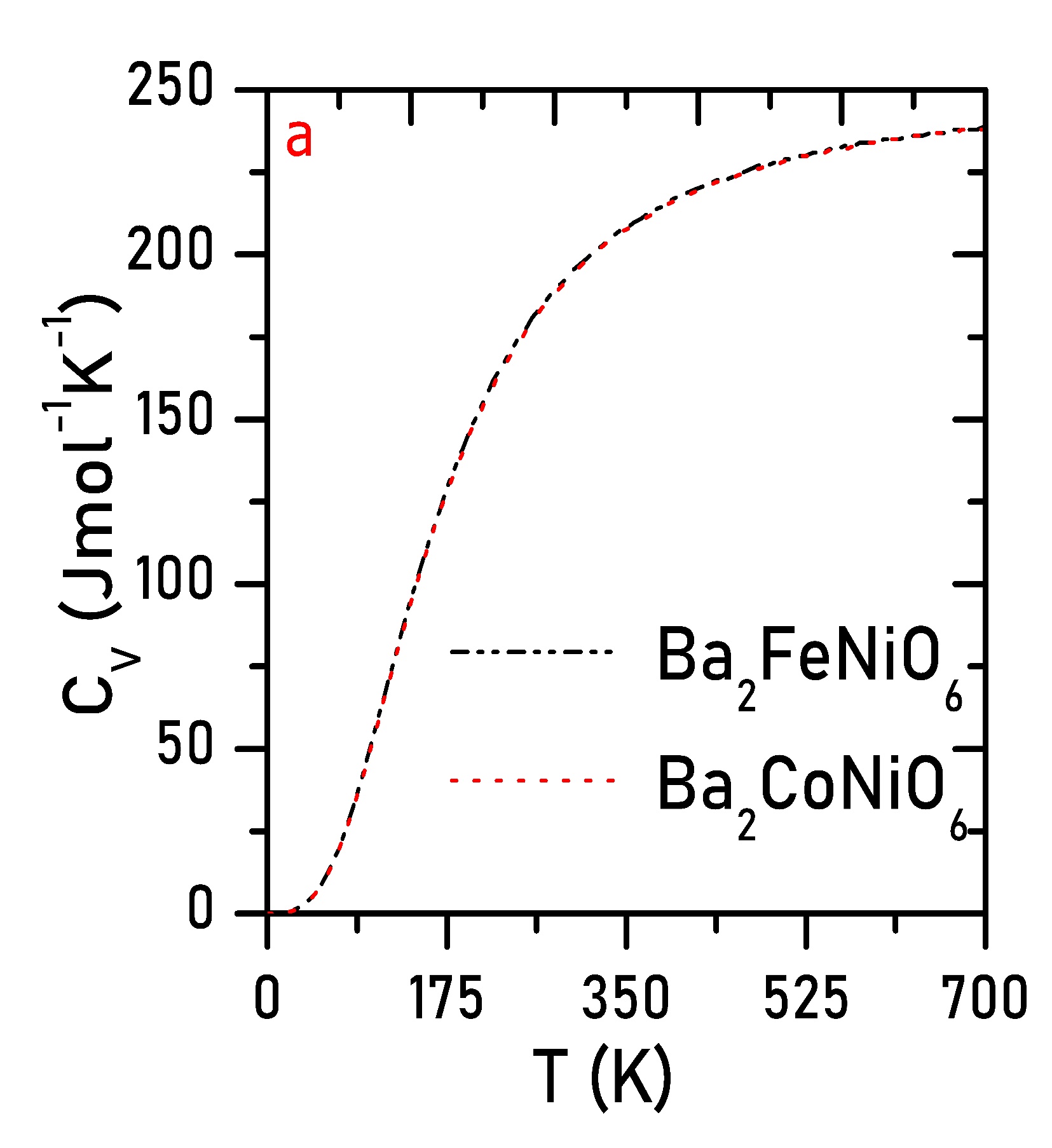


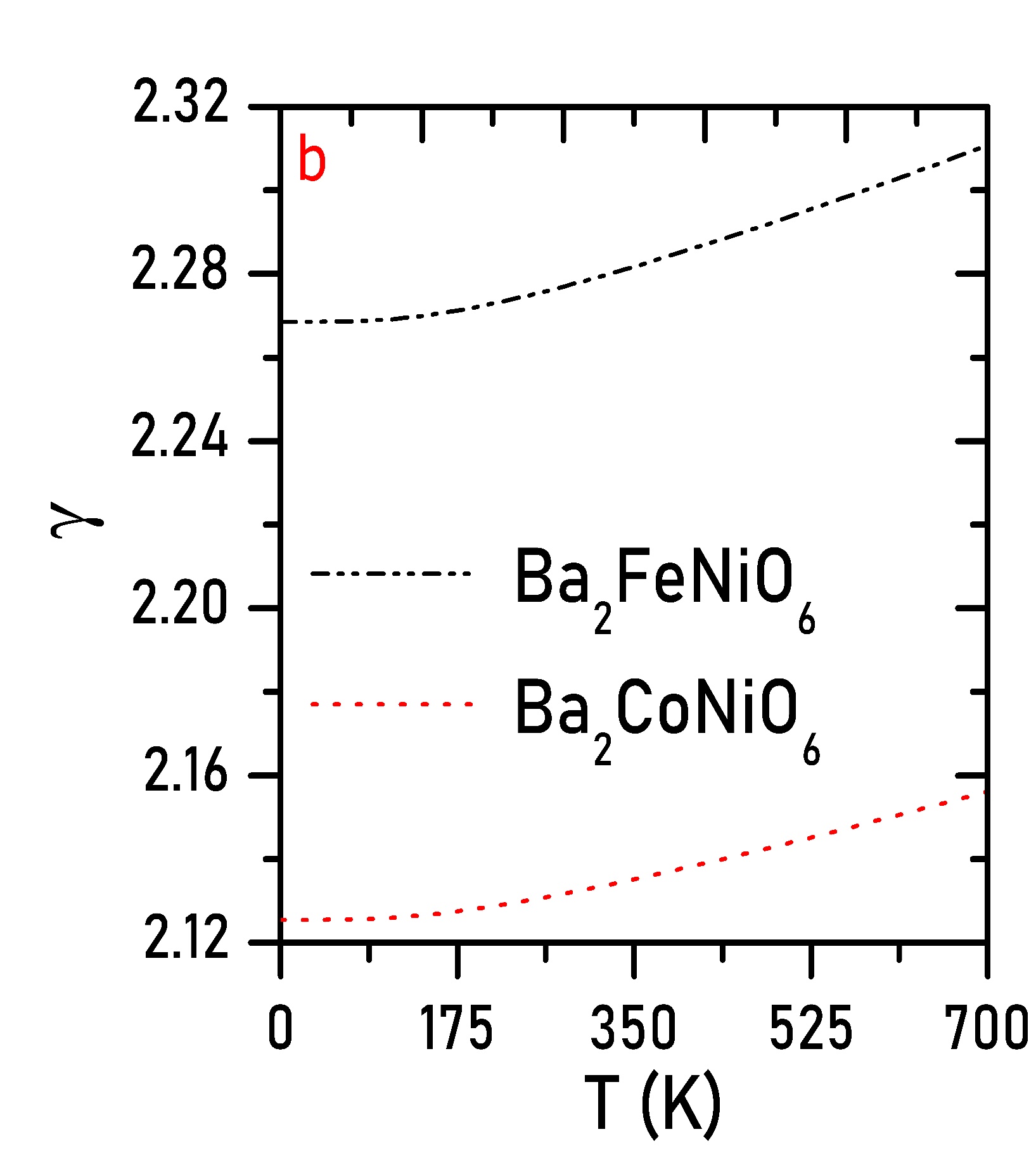


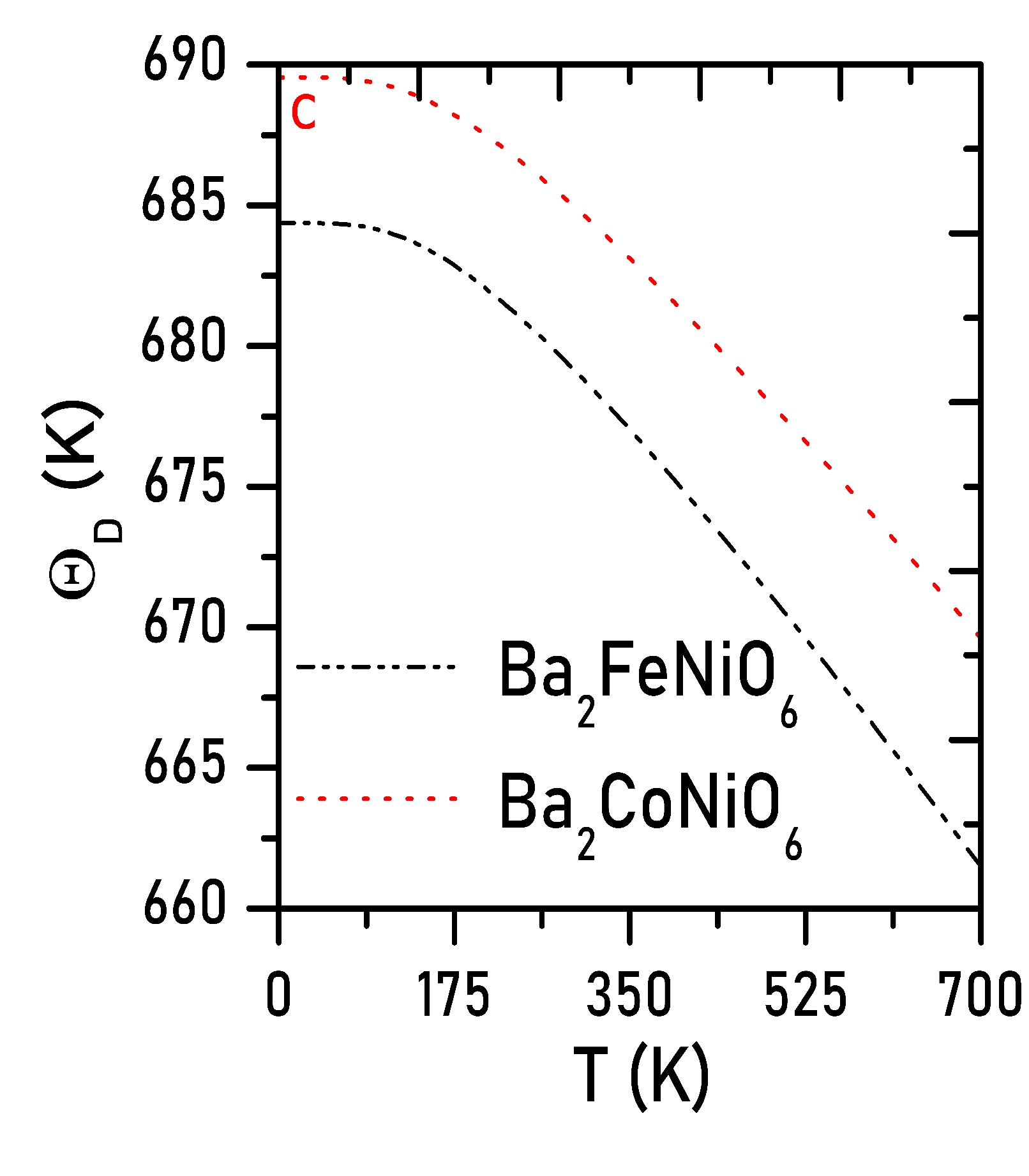


**Fig. S2:** Variation in thermodynamic properties with temperature (a) Specific heat at constant volume; (b) Grüneisen parameter; and (c) Debye temperature

The Grüneisen parameter (γ) is used to measure the anharmonicity in the crystals. **Fig. S2(b)** represents the variations in γ with temperature, advocates rise in anharmonicity increases with the temperature, although the variation is sluggish. The atomic vibrations increase vigorously with temperature due to which γ-parameter also increases. Debye temperature (θ_D_) is one of the important thermo-elastic parameters of solids. It is a good indicator of the hardness; materials with high θ_D_ exhibit are comparatively rigid over materials with low θ_D_. The θ_D_ as a function of temperature is shown in **Fig. S2(c)**, it is clear that θ_D_ decreases with temperature. At the low temperature the thermal expansion, as well as anharmonicity, is small resulting in almost constant Debye temperature. Also, the high-frequency modes can be considered to be frozen at low temperatures. The high value of Debye temperature suggests the perovskites can be used even at higher temperatures for application purposes.

**Electronic Properties**


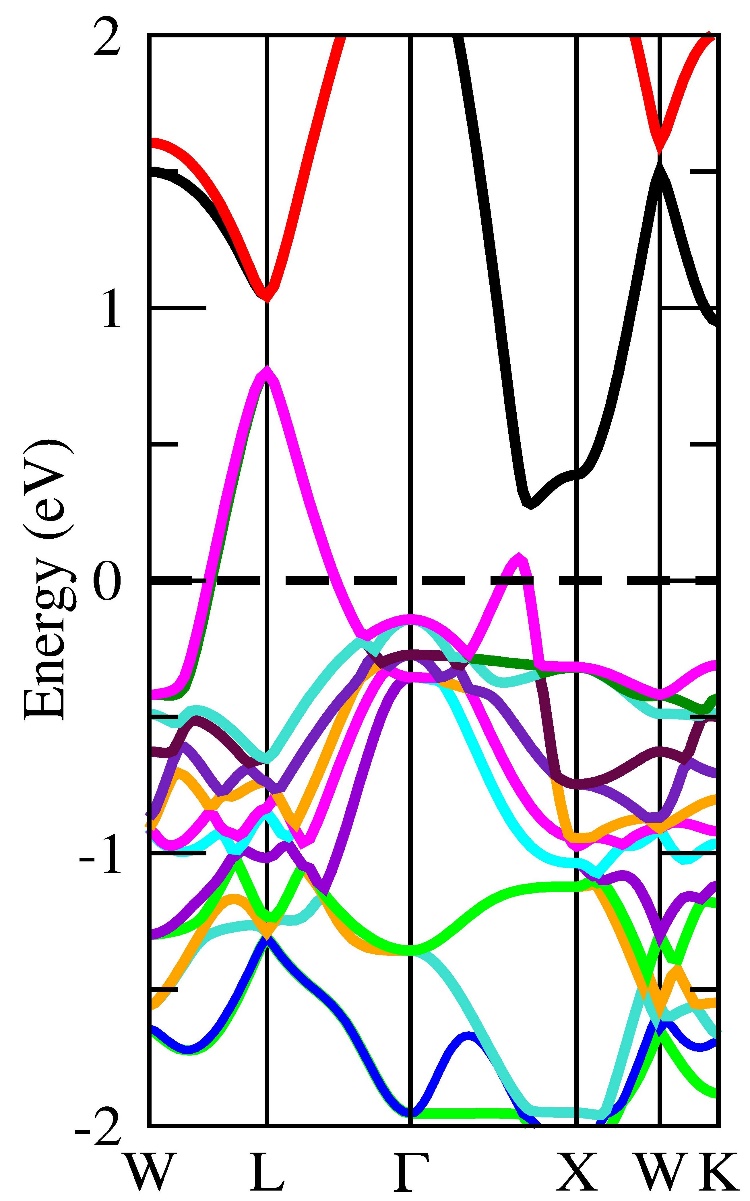

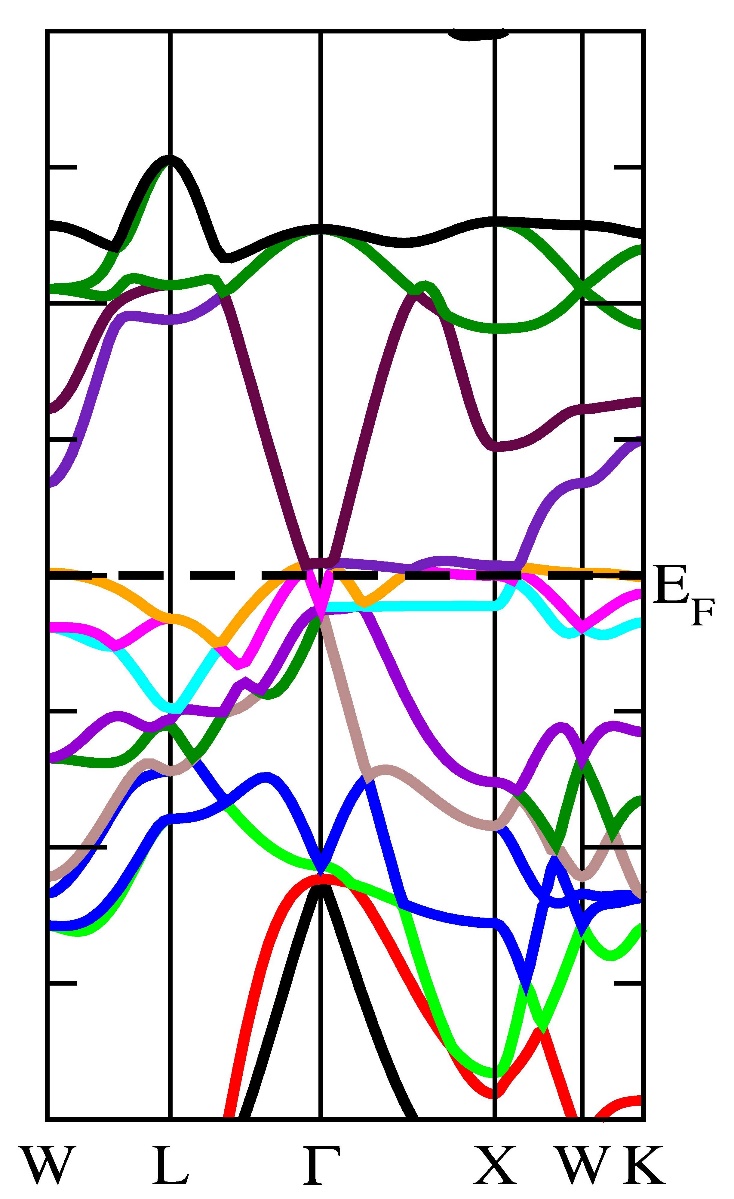


Ba_2_FeNiO_6_


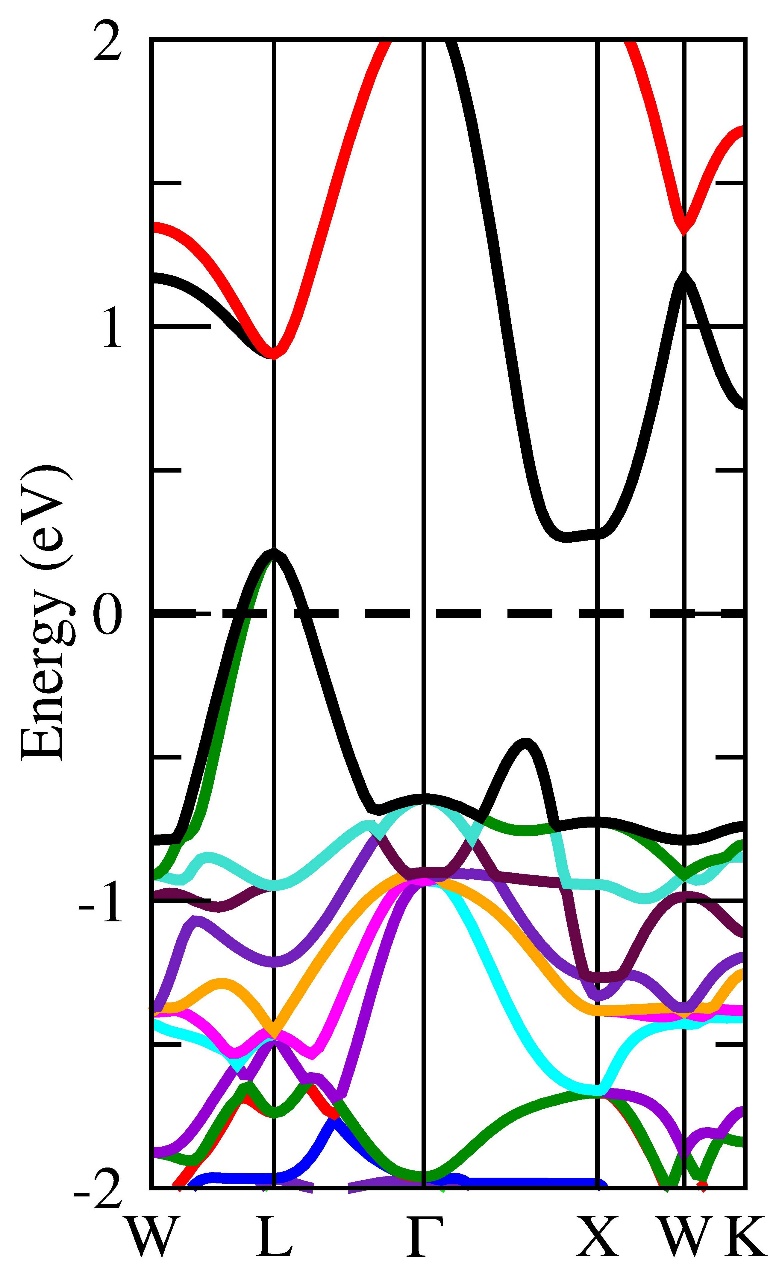

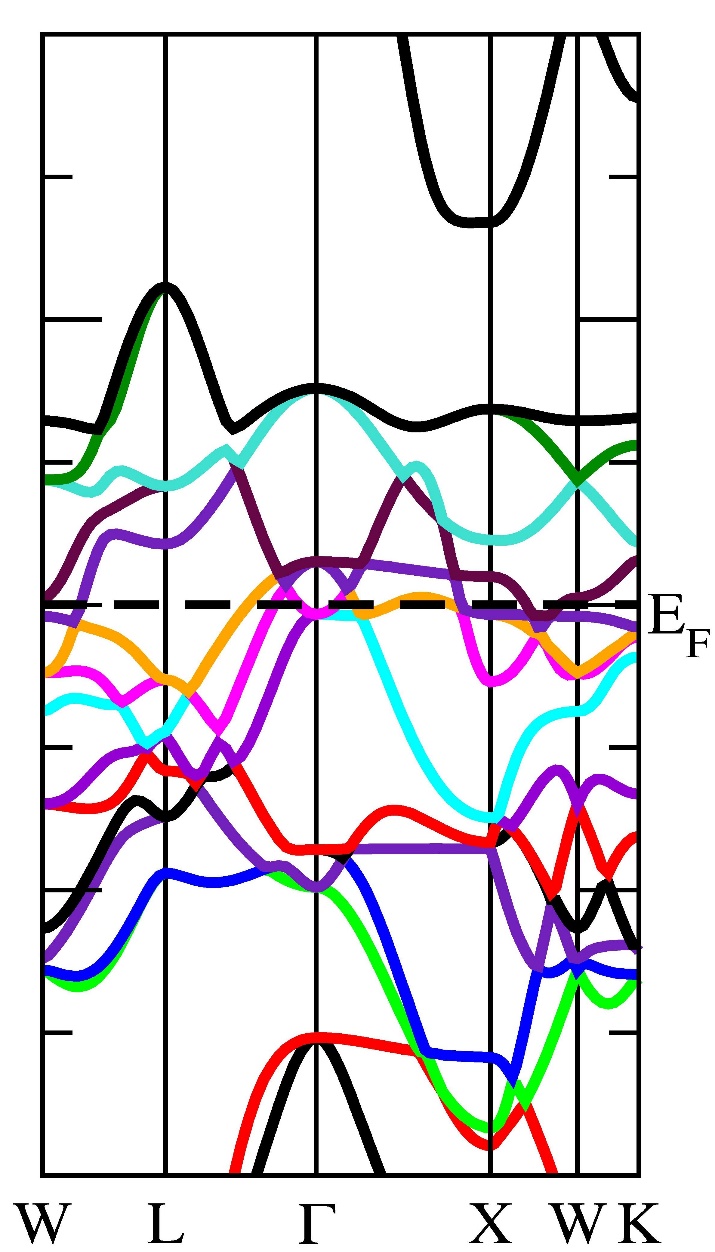


Ba_2_CoNiO_6_

**Fig. S3:** Band Structure of Ba_2_BNiO_6_ computed by applying GGA approximation; passing of Fermi level in-between of bands reflects metallic character (the arrows are used to designate spin channels).

**Fig. S4:** Atomic states contribution towards band formation (a) Ba_2_FeNiO_6_; (b) Ba_2_CoNiO_6_. The transition-metal *d*-states alongwith O-*p* states are more prominent in characterizing the electronic band structure.


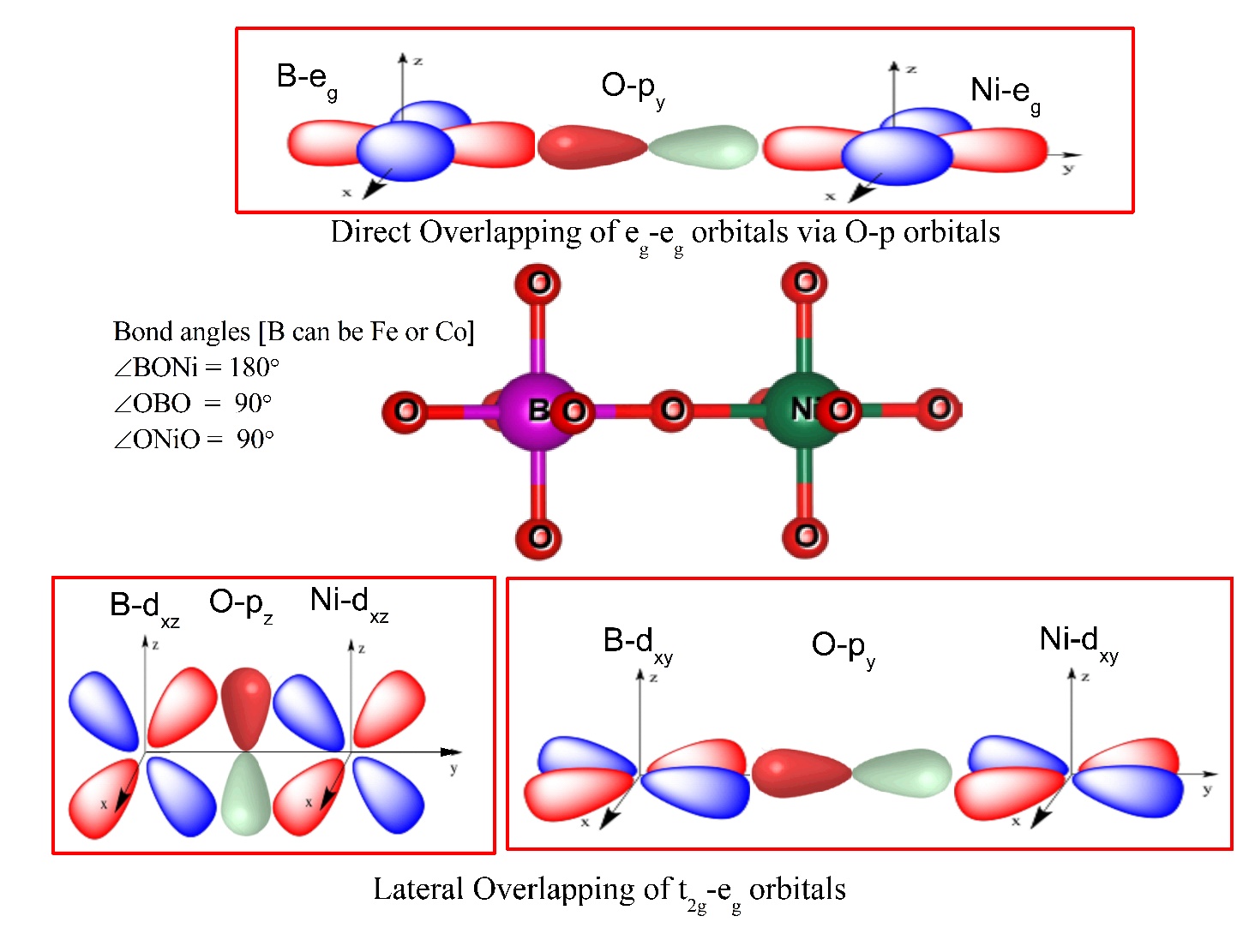
**Fig. S5:** Schematic representation of *d-d* hybridization. The *e_g_*-states of Fe and Co directly head toward the *e_g_*-states of Ni. The *e_g_-e_g_* states thus hybridize via oxygen anion. While the *t_2g_*-states of the transition atoms can only hybridize laterally with the *e_g_* or *t_2g_* states of the other transition atom.

**Thermoelectric Properties**


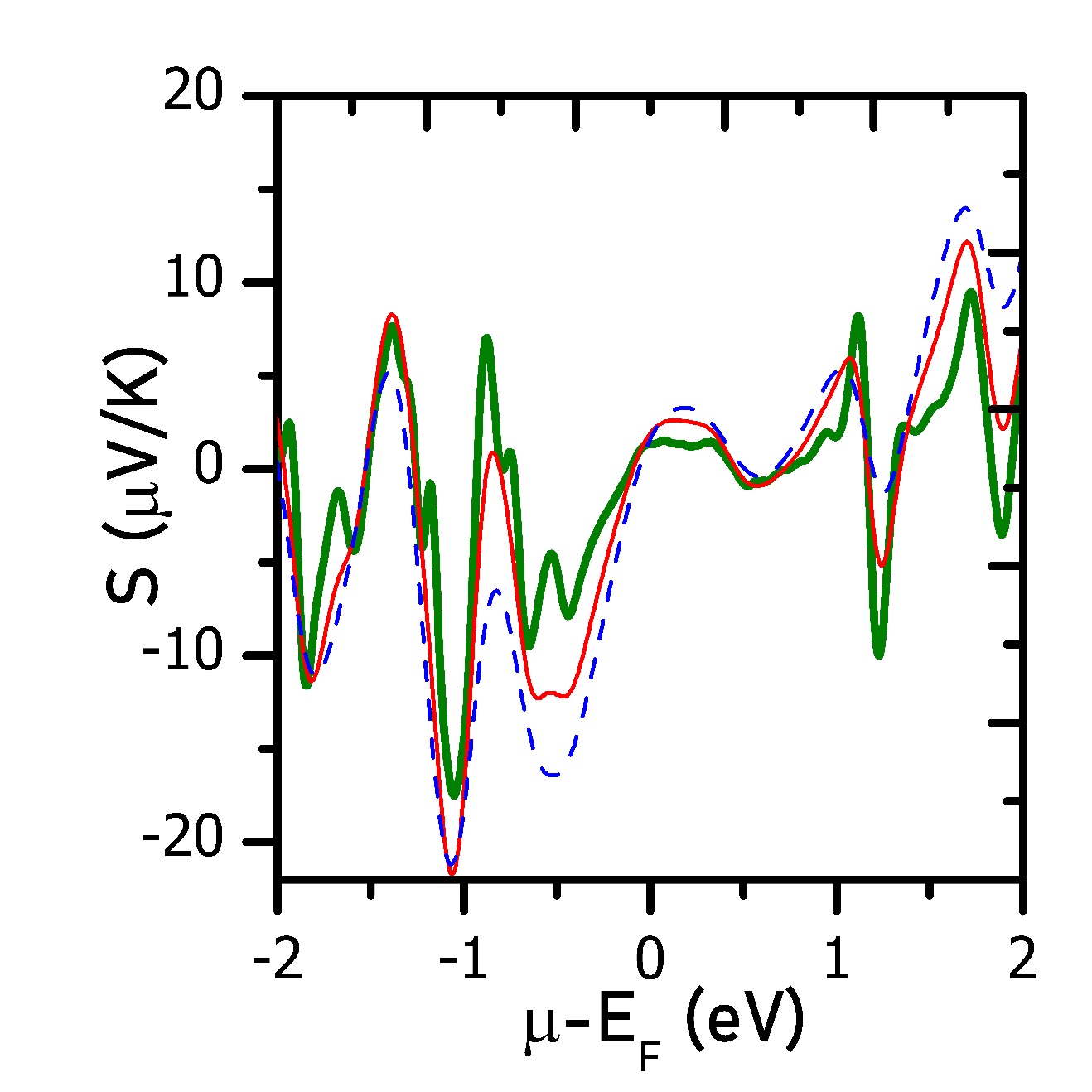

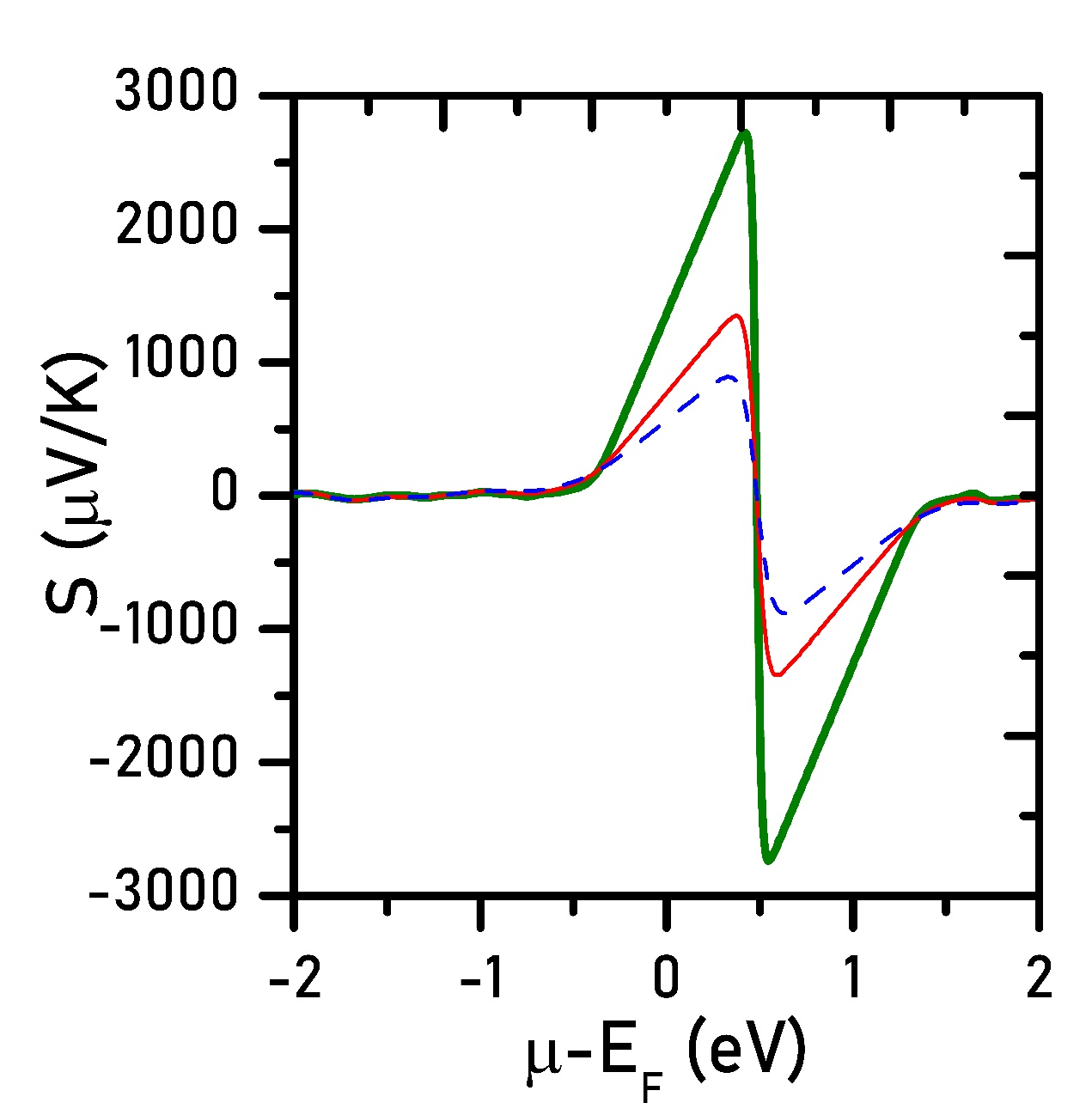


**(a)**


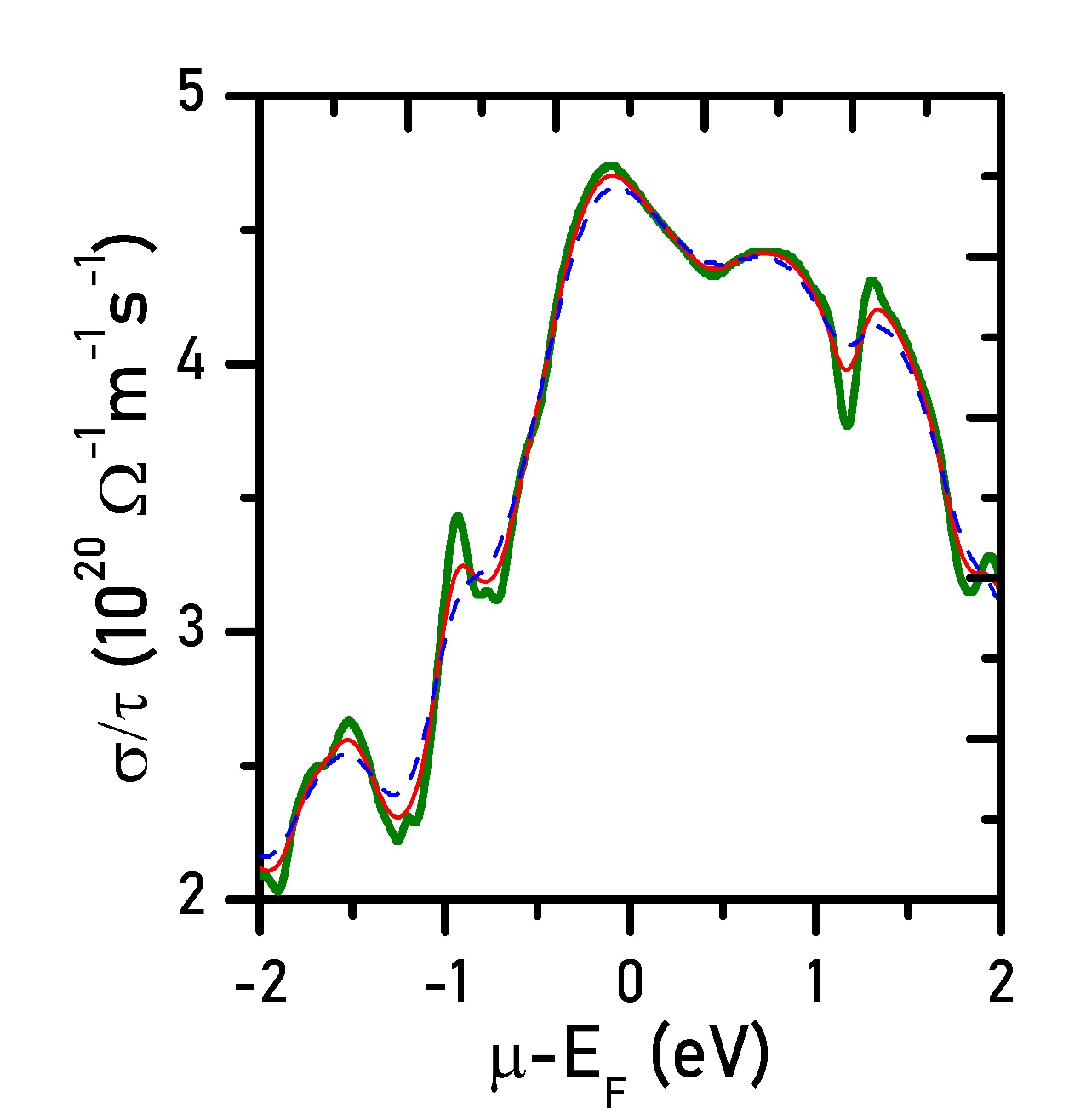

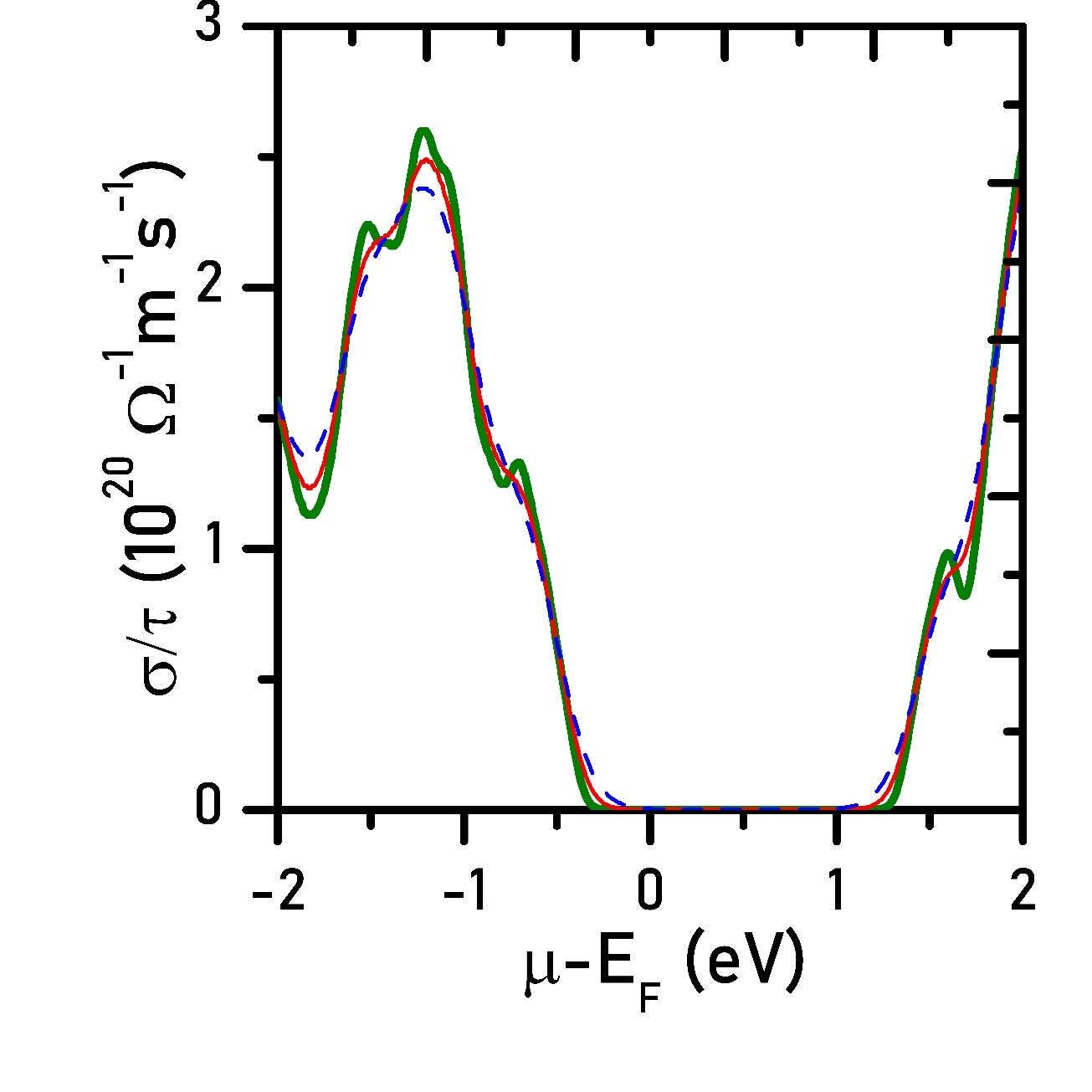


**(b)**

**(c)**


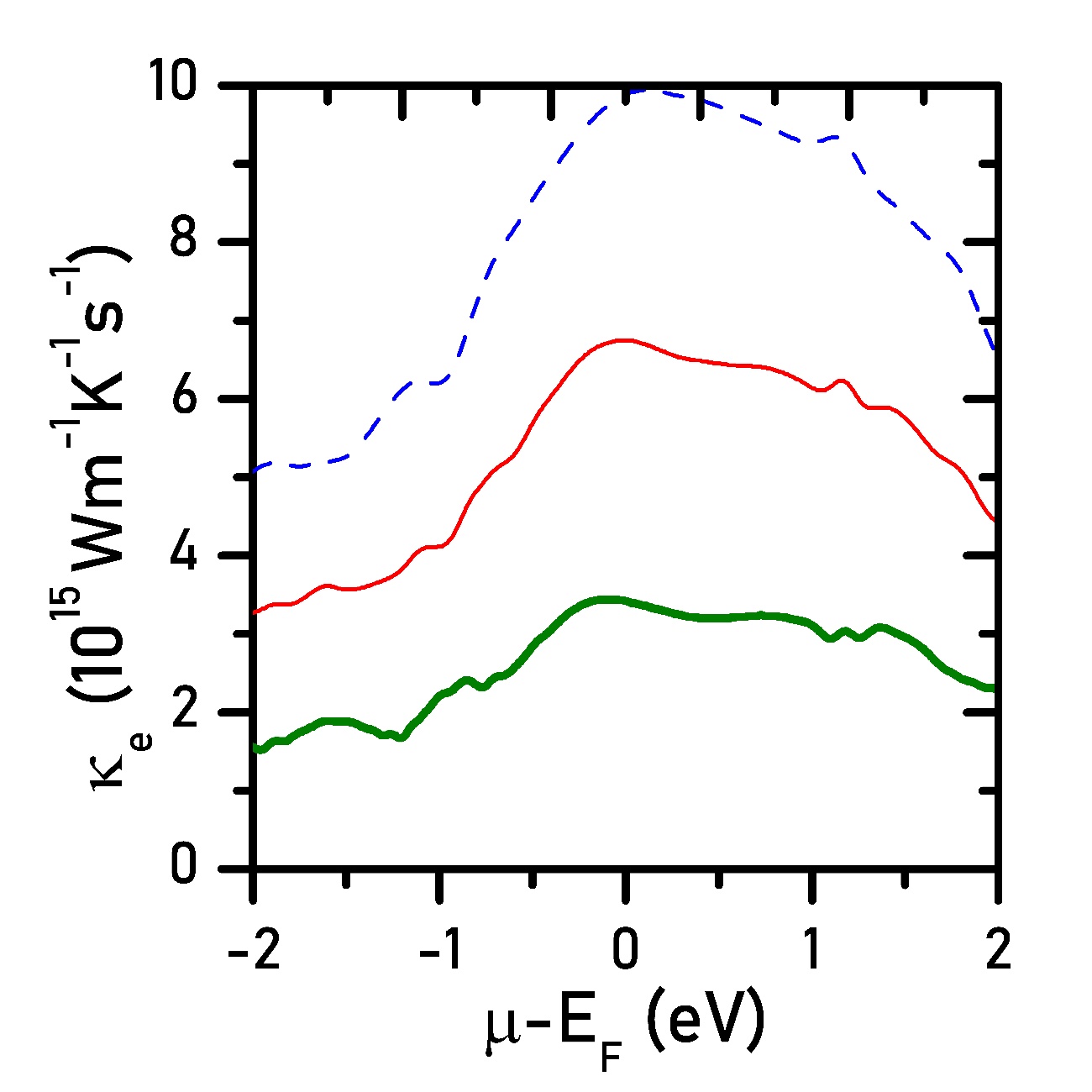

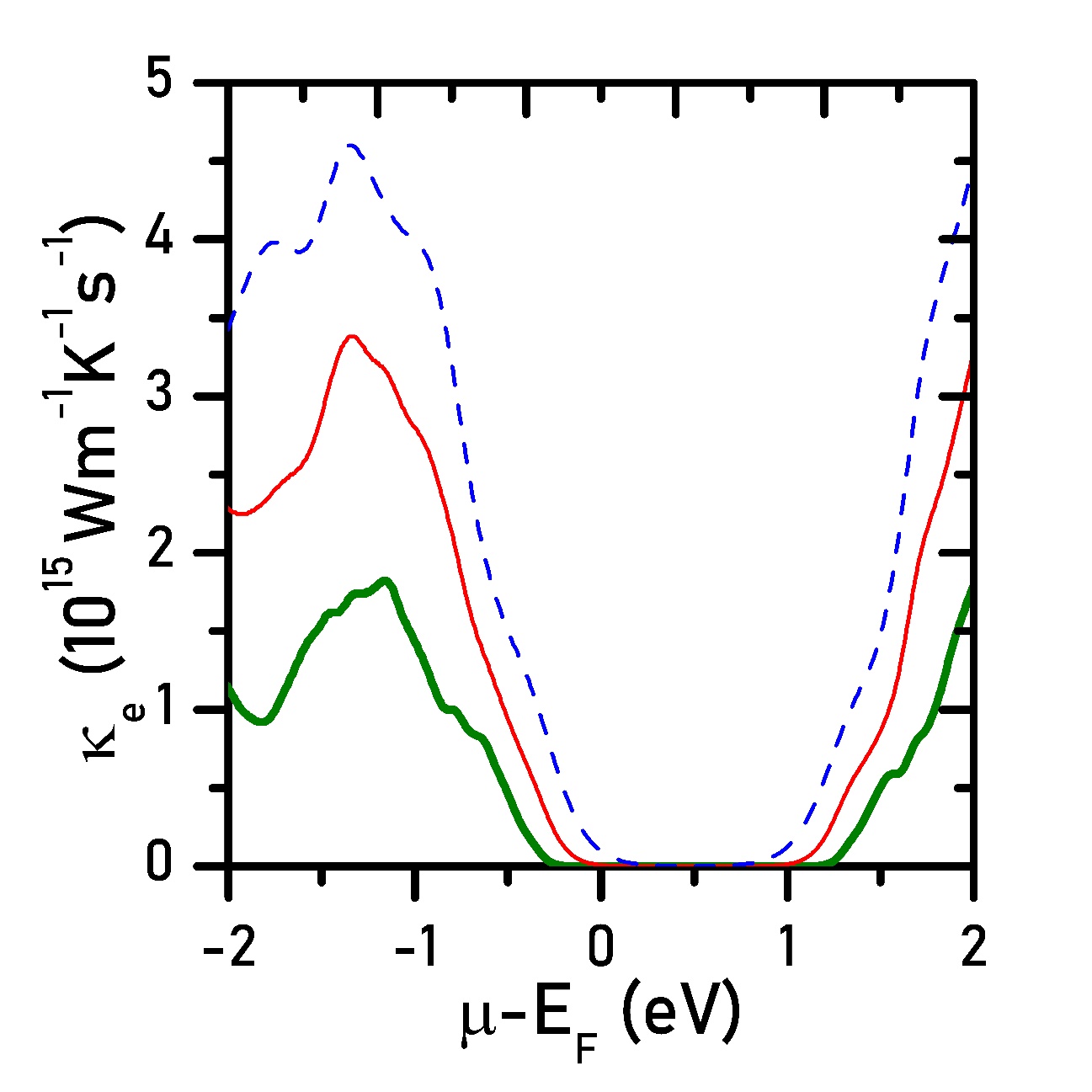


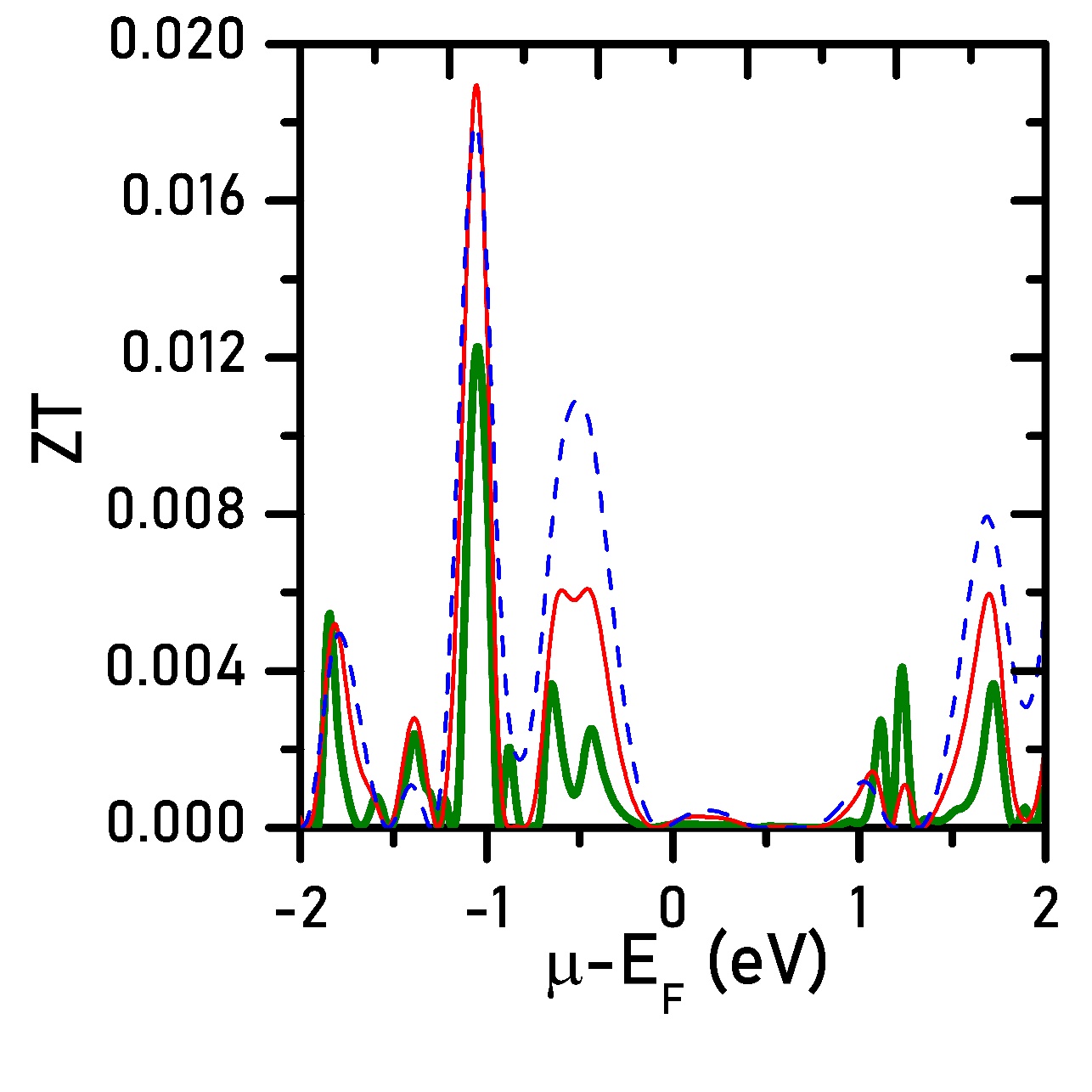

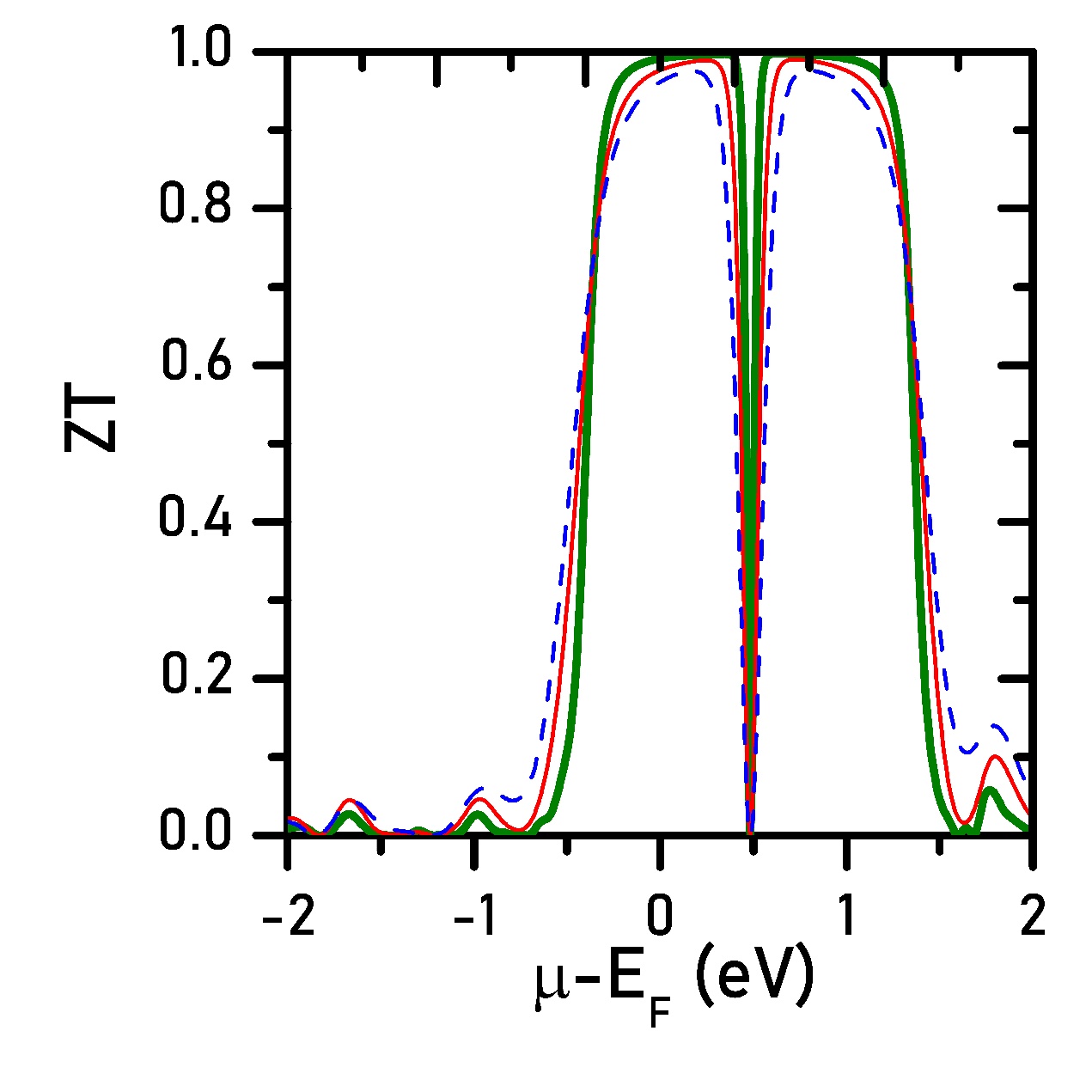


**(d)**

**Fig. S6:** The variation in the designated thermoelectric parameters (a) Seebeck coefficient; (b) Electrical conductivity; (c) Thermal conductivity; (d) Figure of merit of Ba_2_FeNiO_6_. (Arrows are used to represent spin up and spin down channels; different line colors and line styles are used to distinguish temperature Olive (solid thick)-300K, Red (solid thin)-600K, and Blue (dashed)-900K

**d**


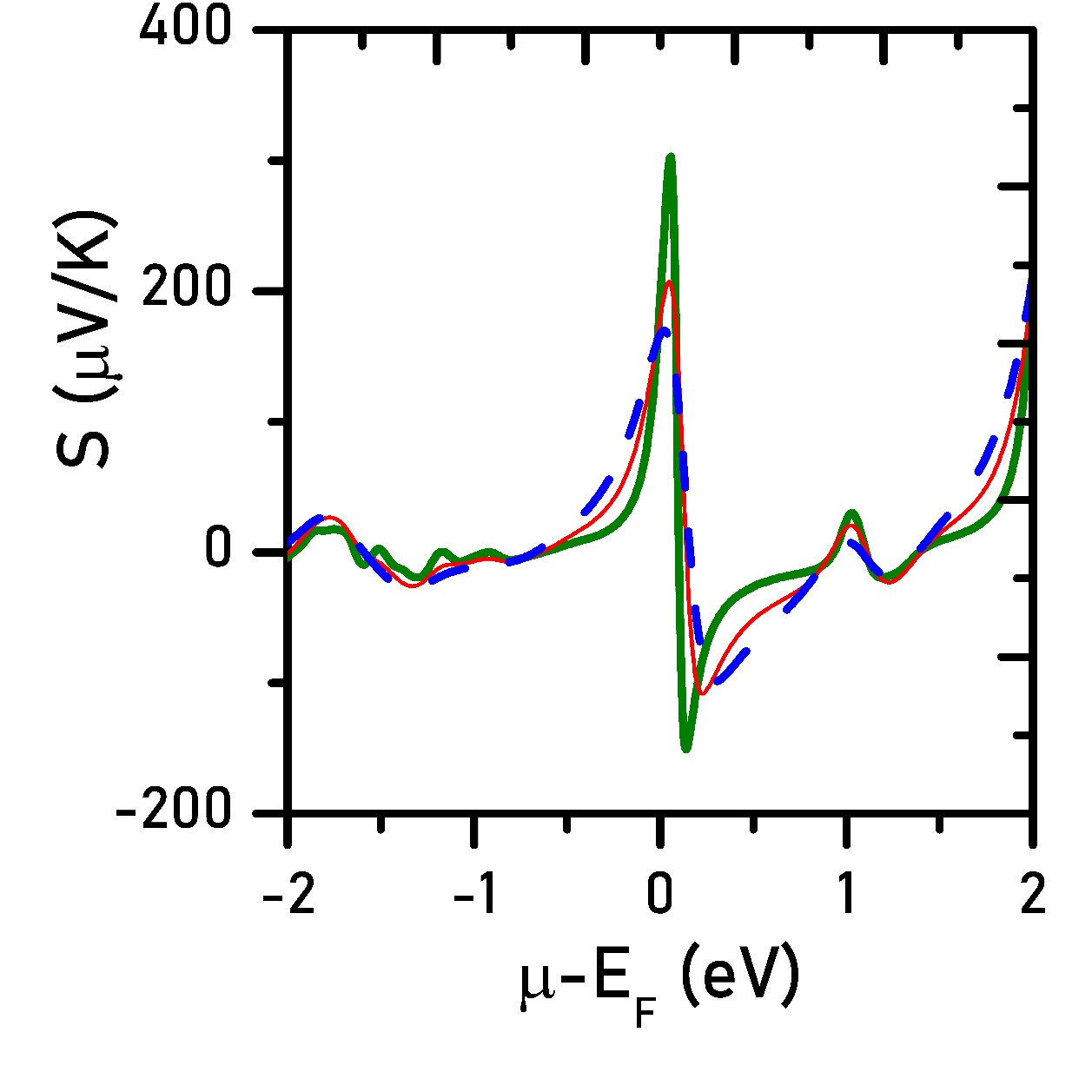

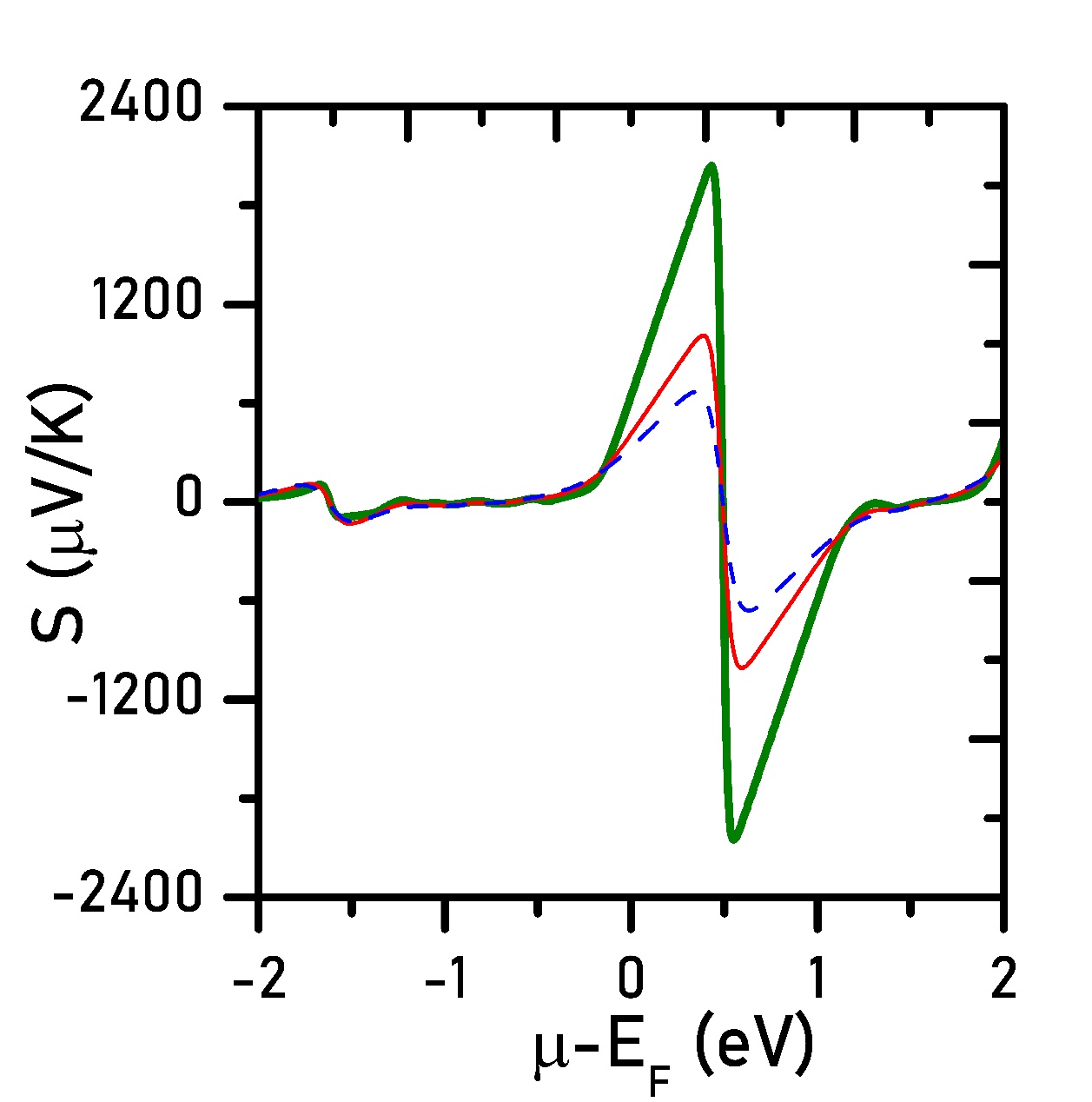


**(a)**

**(b)**


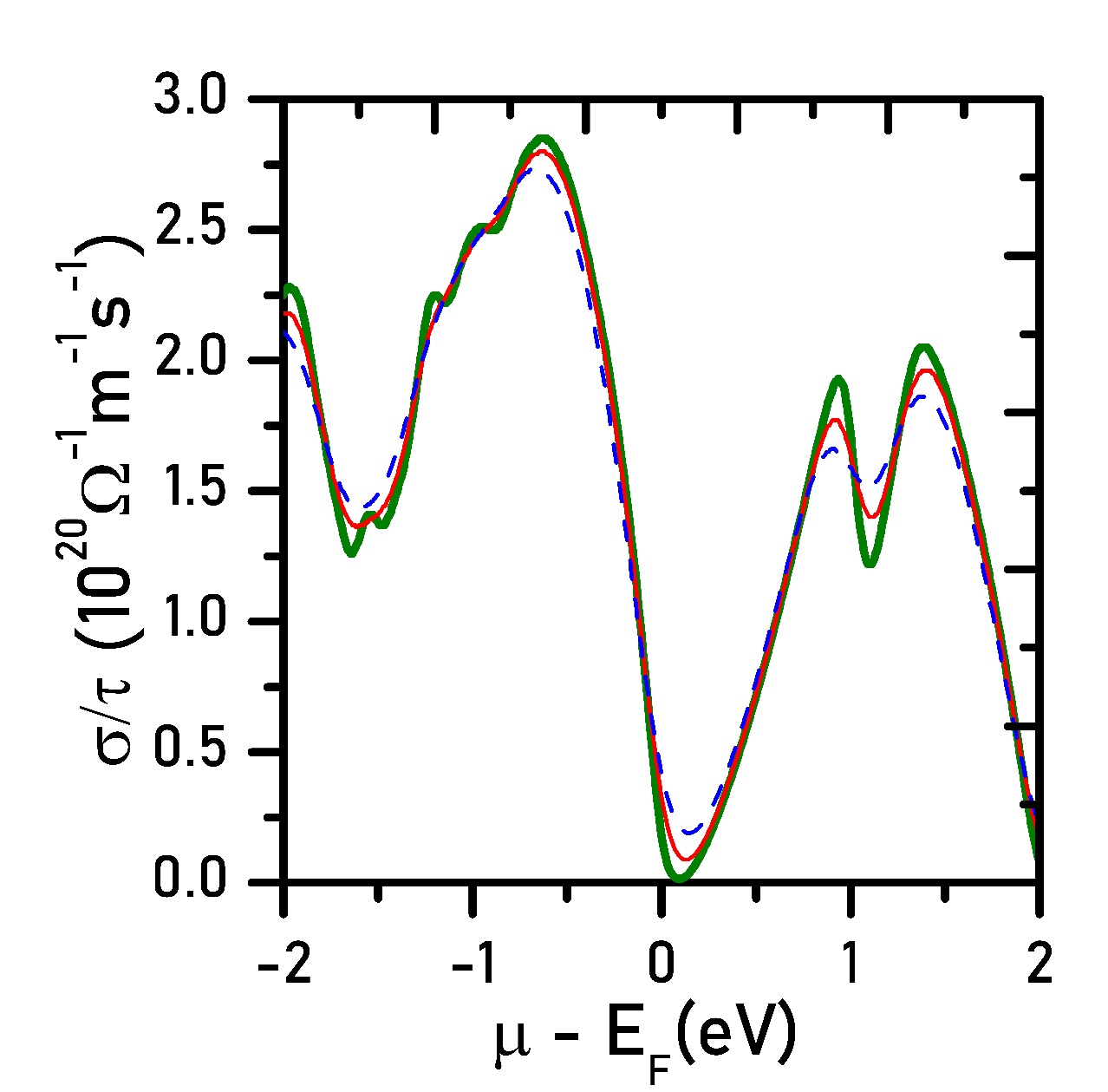

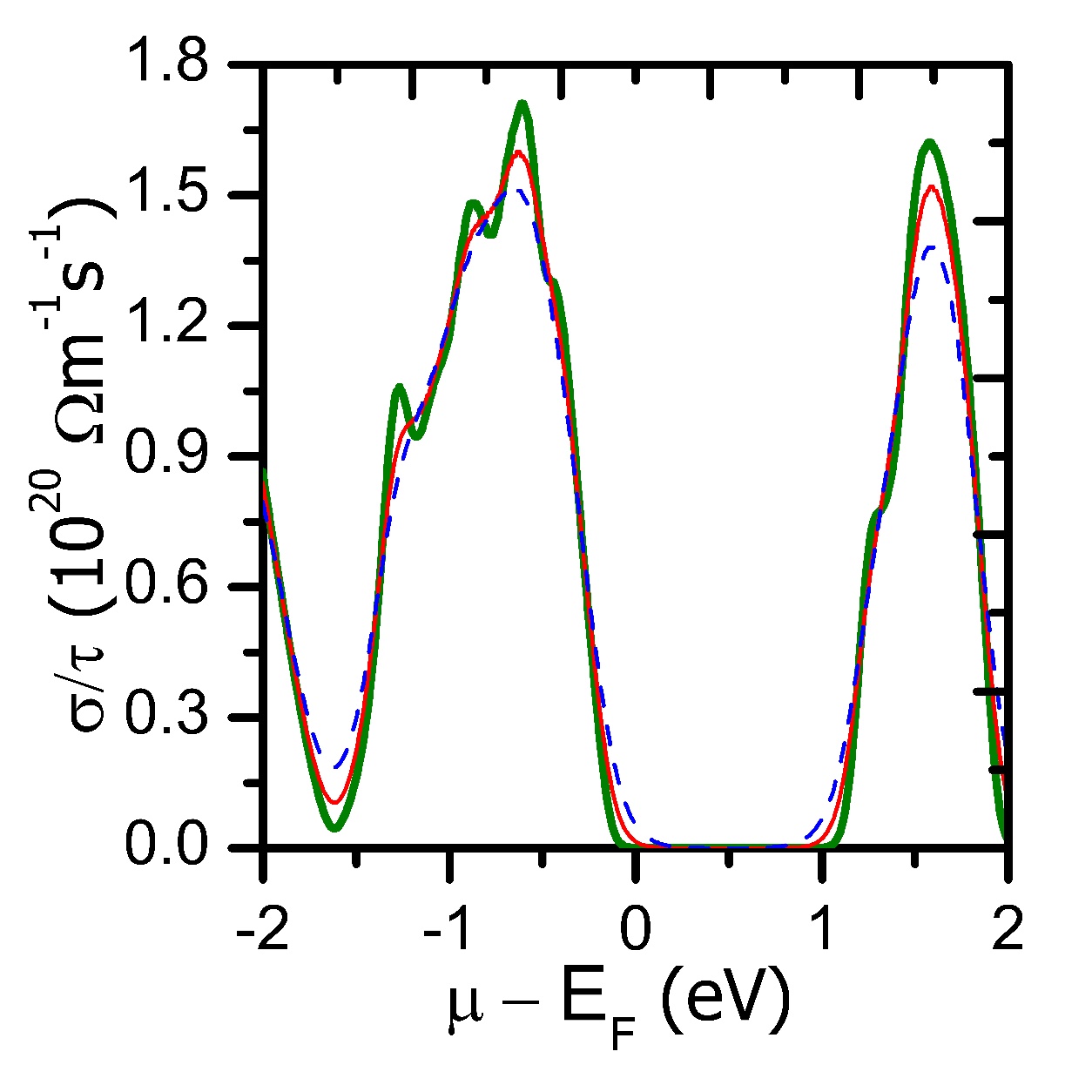


**(c)**


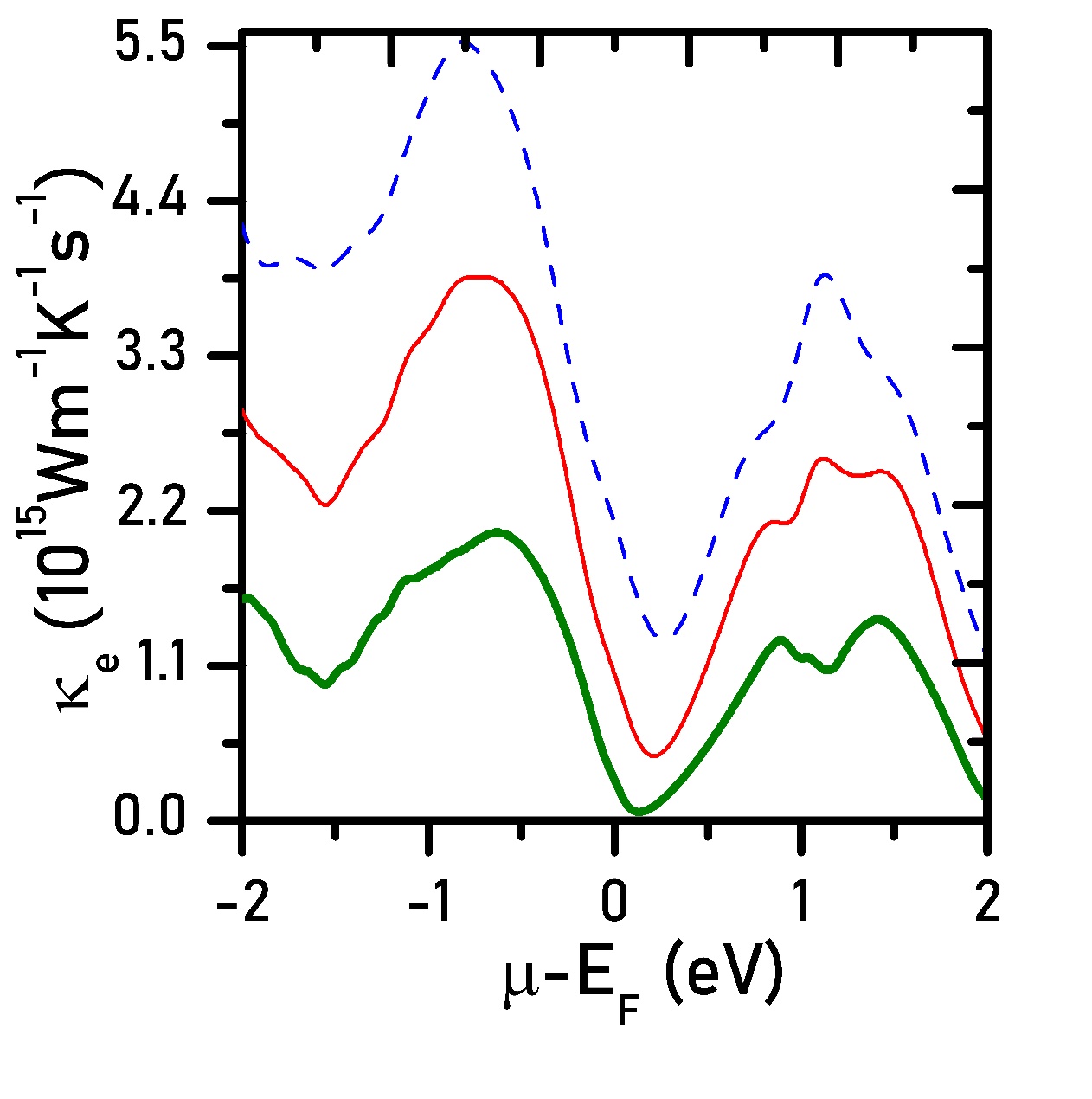

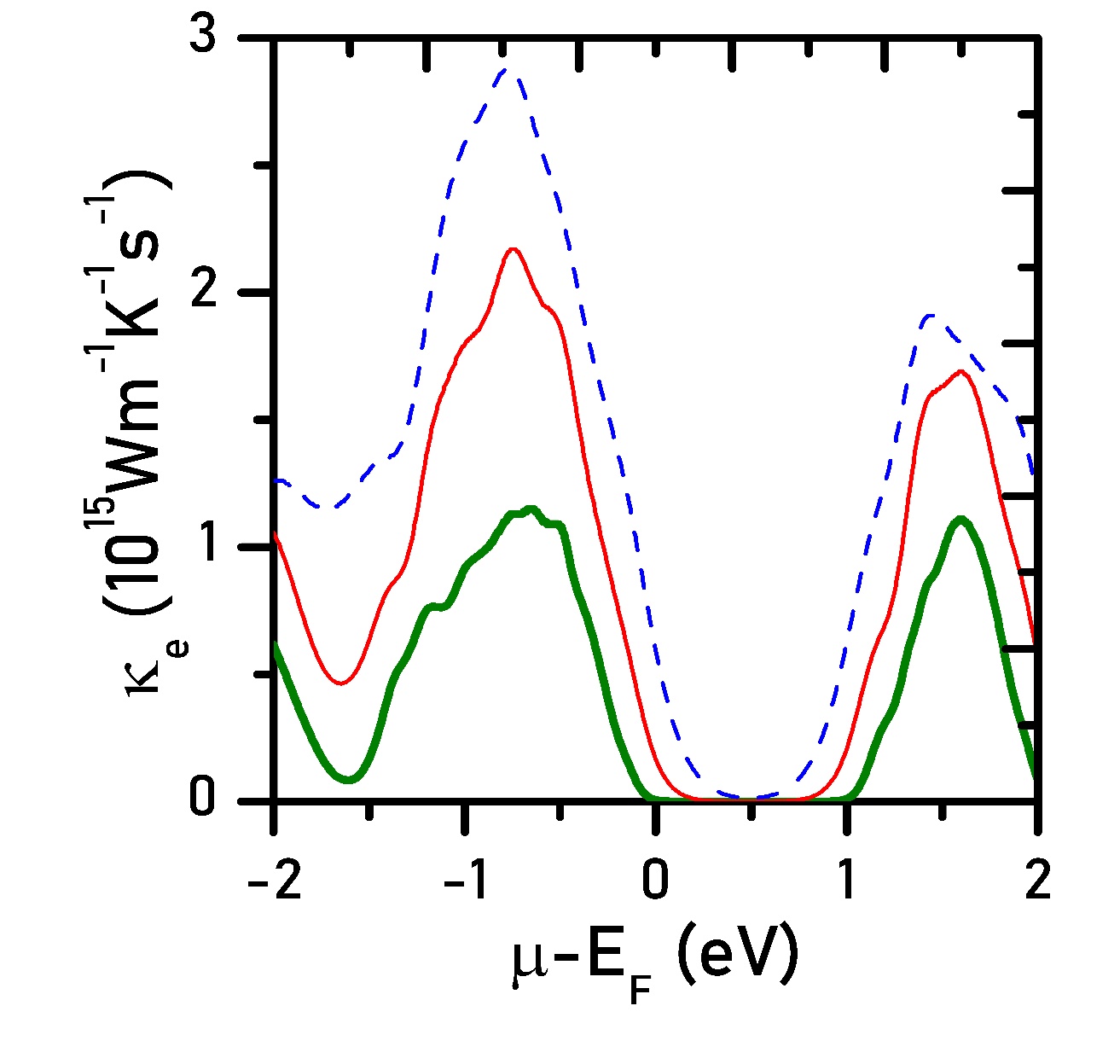


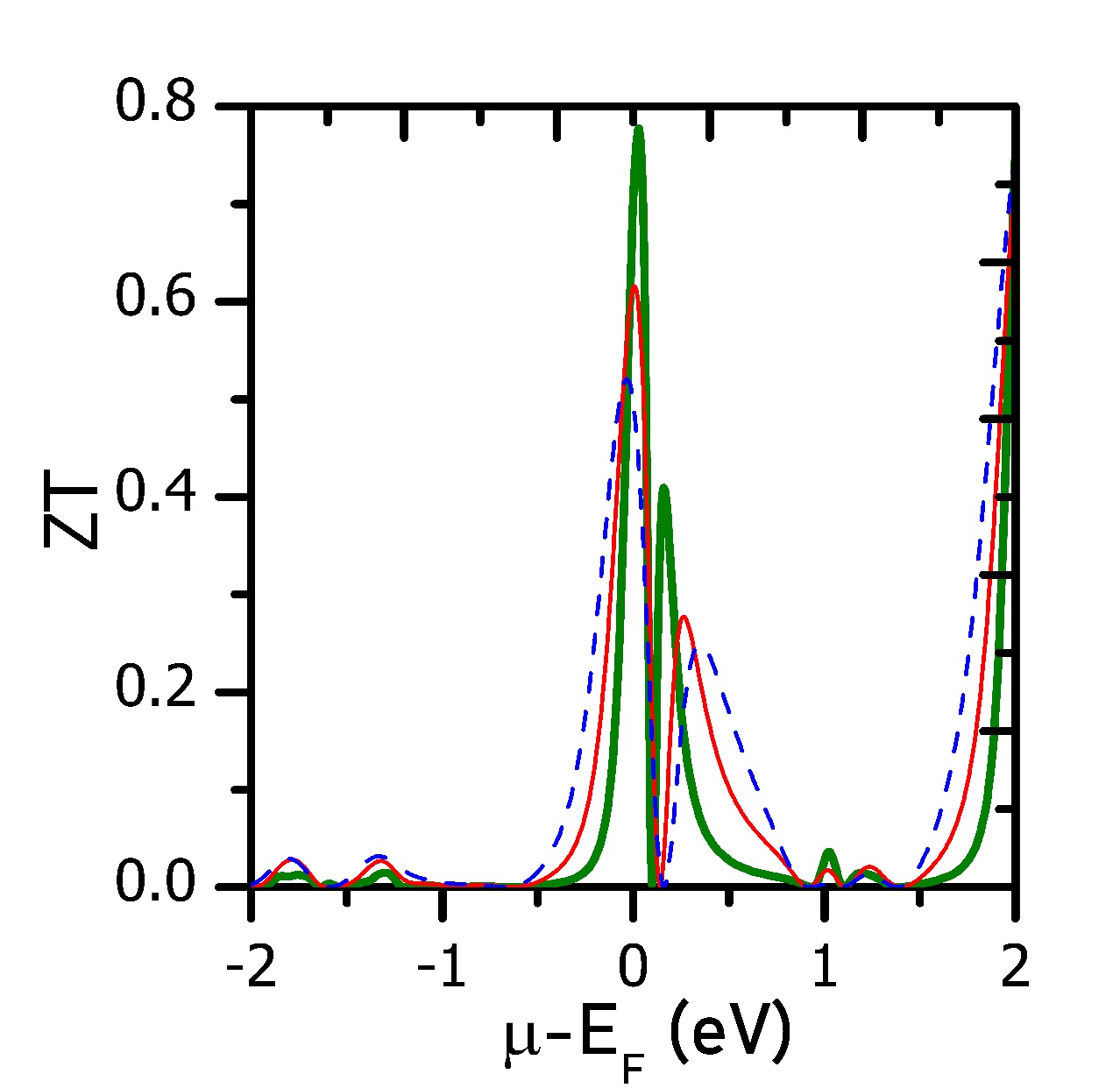

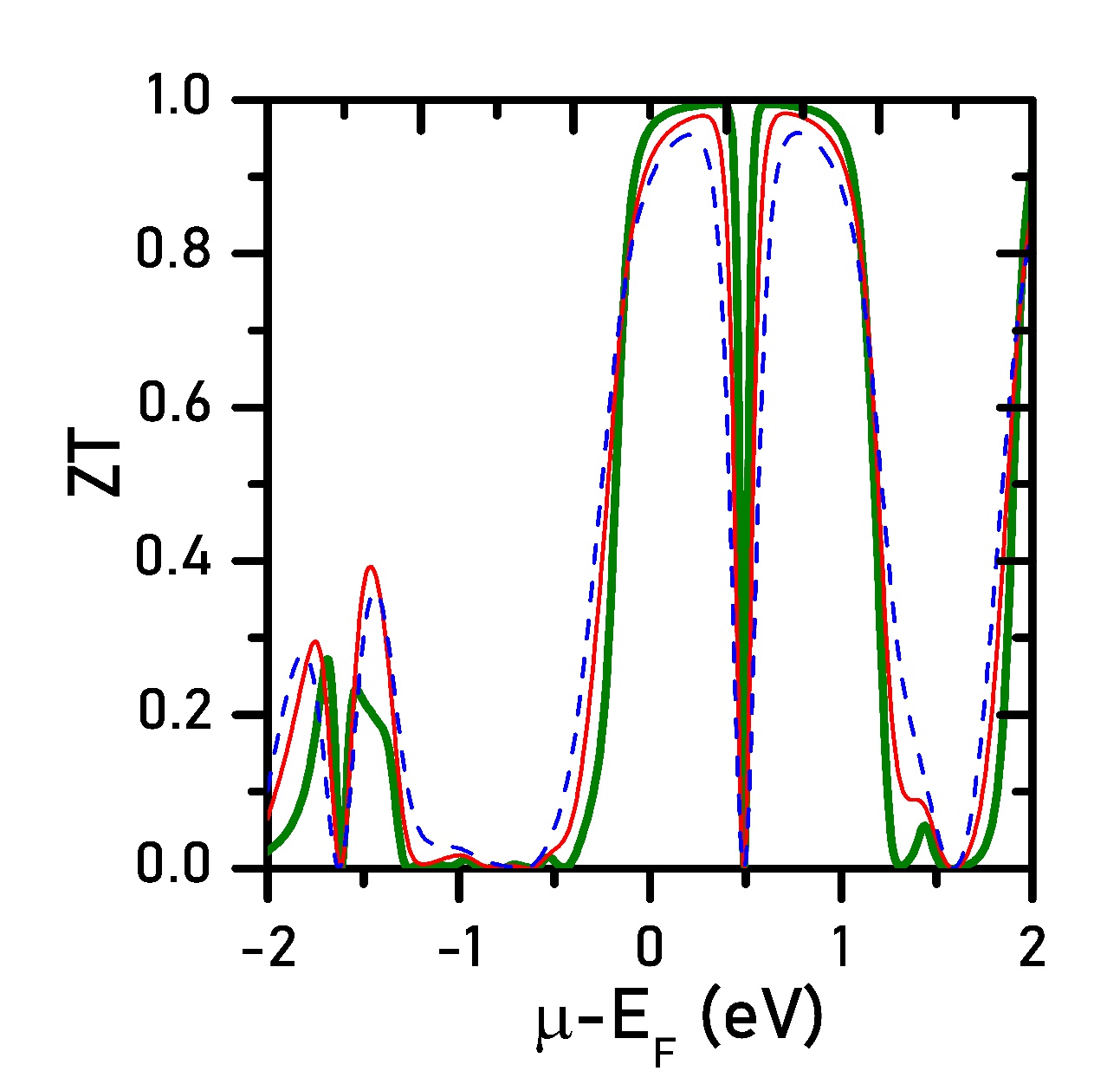


**(d)**

**Fig. S7:** The variation in the designated thermoelectric parameters (a) Seebeck coefficient; (b) Electrical conductivity; (c) Thermal conductivity; (d) Figure of merit of Ba_2_CoNiO_6_. (Arrows are used to represent spin up and spin down channels; different colors and line styles are used to distinguish temperature: Olive (solid thick)-300K; Red (solid thin)-600K; and Blue (dotted)-900K

The change in the behavior of transport properties from spin-up to spin-down channel is shown in **Fig. S6** is expected due to metallic behavior in the spin-up channel and the semiconducting nature in the spin-down channel. The figure of merit (ZT) varies inversely with thermal conductivity. Due to the metallic nature of the spin-up channel, the thermal conductivity is high thereby ZT is very low. However, in the spin-down channel, there is vanishing thermal conductivity around the Fermi level, which overturns the ZT ~1. However, the total thermal conductivity, as well as electrical conductivity, is the sum of two channels, due to which the metallic channel remains more dominant in characterizing the overall thermoelectric behavior of the half-metallic materials.

Ba_2_CoNiO_6_ is a ferromagnetic semiconductor with a small bandgap in the spin-up channel compared to spin down the channel. The *e_g_*-states of transition metals are filled in spin up-channel lie beneath Fermi level gives rise to a small bandgap. However, due to the semiconducting nature, the thermal conductivity is low compared to the Fe-based counter-partner (see **Fig. S7**). Because of that ZT in both spin channels as well as total ZT turns to be ~0.8. However, with the rise in temperature energy bands smear give a metallic effect in the small bandgap spin channel, so ZT decreases drastically.

**Fig. S8:** Comparative variation in carrier concentration (n), Seebeck coefficient (S), and electric conductivity over relaxation time (σ/τ) along with the volumetric density of states (DOS) at 300K with the chemical potential for Ba_2_CoNiO_6_. (Arrows are used to describe spin channels).

The relation of Seebeck coefficient with carrier concentration in case of simple metals or degenerate semiconductors with parabolic bands is; $S=\frac{8\pi^{2}k_{B}^{2}}{3e\hbar^{2}}m^{*}T\left( \frac{\pi}{3n} \right)^{2/3}\text{, }$ implies magnitude of ‘S’ decrease with an increase in ‘n’ ^[7]^. On the other side, conductivity increases with the rise in effective charge carries. The graphical variation **Fig. S8** signifies ‘n’ increase with the rise in DOS peaks on either side of Fermi level at 0 eV, but exhibit sharp(flat) variation over the bandgap region. The negative sign of carrier concentration above 0 eV signifies electrons are majority carriers in the conduction band and a positive sign is used for holes. So, as the ‘n’ increases on both sides of E_F_ S-decreases sharply, while conductivity increases. The Seebeck coefficient has high values for the forbidden region.

**References**

1. Anisimov, V. I., Solovyev, I. V., Korotin, M. A., Czyżyk, M. T., & Sawatzky, G. A. (1993). Density-functional theory and NiO photoemission spectra. *Physical Review B*, *48*(23), 16929.
2. Yanai, T., Tew, D. P., & Handy, N. C. (2004). A new hybrid exchange–correlation functional using the Coulomb-attenuating method (CAM-B3LYP). *Chemical physics letters*, *393*(1-3), 51-57.
3. Chibani, S., Arbouche, O., Amara, K., Zemouli, M., Benallou, Y., Azzaz, Y., ... & Ameri, M. (2017). A computational study of the optoelectronic and thermoelectric properties of HfIrX (X= As, Sb and Bi) in the cubic LiAlSi-type structure. *Journal of Computational Electronics*, *16*(3), 765-775.
4. Souidi, A., Bentata, S., Benstaali, W., Bouadjemi, B., Abbad, A., & Lantri, T. (2016). First principle study of spintronic properties for double perovskites Ba_2_XMoO_6_ with X= V, Cr and Mn. *Materials Science in Semiconductor Processing*, *43*, 196-208.
5. Yousuf, S., & Gupta, D. C. (2018). Ternary germanide Li2ZnGe: a new candidate for high temperature thermoelectrics. *Journal of Alloys and Compounds*, *738*, 501-508.
6. Mir, S. A., & Gupta, D. C. (2021). Understanding the origin of semiconducting ferromagnetic character along with the high figure of merit in Cs_2_NaMCl_6_ (M= Cr, Fe) double perovskites. *Journal of Magnetism and Magnetic Materials*, *519*, 167431.
7. Reshak, A. H. (2016). Transport properties of Co-based Heusler compounds Co_2_VAl and Co_2_VGa: spin-polarized DFT+ U. *RSC advances*, *6*(59), 54001-54012.
